# Spatially Explicit Assessment of Pathogen-Related Health Impacts in Life Cycle Assessment

**DOI:** 10.1021/acs.est.6c10006

**Published:** 2026-09-16

**Authors:** Vanessa Schenker, Yao Zhou, Stephan Pfister

**Affiliations:** 27219 Swiss Federal Institute of Technology Zurich, Chair of Ecological Systems Design, Laura-Hezner-Weg 7, CH - 8093 Zurich, Switzerland

**Keywords:** life cycle assessment, quantitative microbial risk assessment, waterborne pathogens, wastewater treatment, disability-adjusted life years, recreational water exposure, spatial modeling, regionalized characterization factors

## Abstract

Pathogen-related human health impacts from wastewater emissions are not normally addressed in life cycle impact assessment (LCIA), limiting the use of life cycle assessment (LCA) to compare wastewater infrastructure and management options that differ in treatment performance, discharge location, and exposure context. Building on quantitative microbial risk assessment (QMRA), which has identified recreational water use as an important exposure pathway for wastewater-related pathogens, this study translates QMRA into a spatially explicit LCIA framework for pathogen emissions to surface waters. The framework links wastewater treatment plant emissions, pathogen fate in rivers and lakes, recreational exposure at bathing sites, dose–response relationships, and disability-adjusted life years (DALYs) to derive characterization factors (CFs) compatible with LCA. In a national-scale application to Switzerland, we assess six wastewater-related pathogens transported through the river and lake network to 503 designated bathing sites. The estimated annual health burden is 1150 DALYs, with strong spatial concentration: the top 10 bathing sites account for 64 % of the total burden. Basin- and pathogen-specific CFs span up to five orders of magnitude, showing that generic or spatially averaged factors can obscure decision-relevant variation. The framework provides the first pathogen-specific CFs for wastewater emissions to surface waters and enables pathogen-related human health impacts to be integrated into LCA.

## Graphical abstract

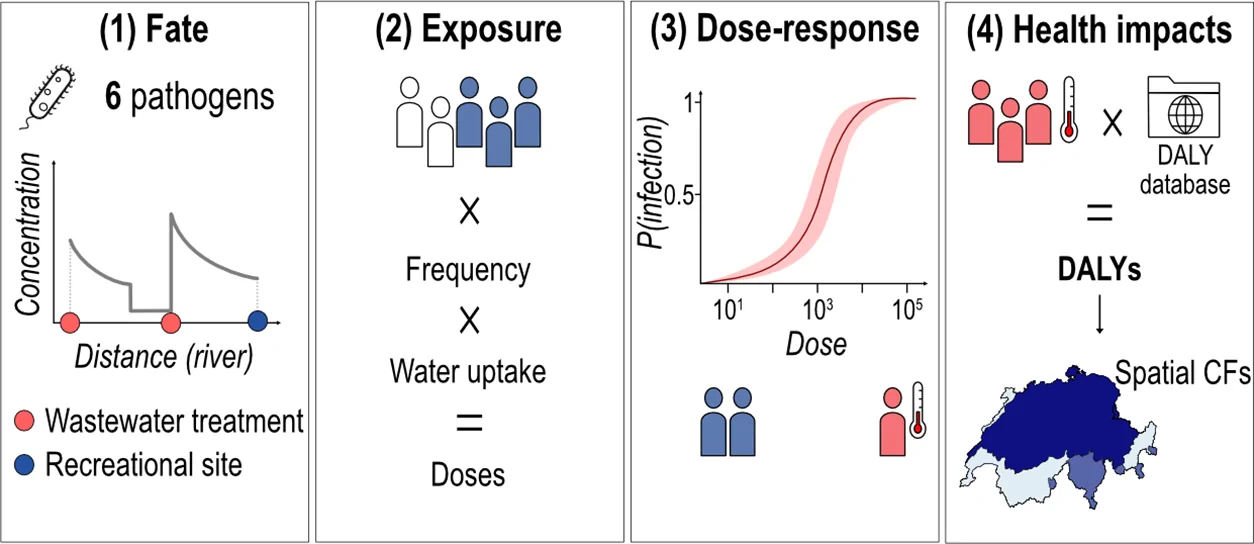

## Introduction

Given the substantial environmental impacts of wastewater infrastructure and management, life cycle assessment (LCA) is increasingly applied to support decision-making and to compare options from an environmental perspective. However, existing life cycle impact assessment (LCIA) methods do not capture human health impacts caused by pathogenic microorganisms. Yet pathogens originating from wastewater remain a persistent public health concern, even in regions with advanced wastewater treatment. Wastewater effluents introduce microbial contamination into surface waters, and extreme events such as flooding or heavy rainfall can further elevate pathogen loads.^1–4^ In the context of climate change, the frequency and intensity of such events are expected to increase, intensifying the need for robust and forward-looking wastewater management strategies and to holistically assess their performance.^2,3,5^

Effective decision-making in wastewater management requires accounting for human health risks arising from pathogen exposure in the receiving waters. It also requires evaluating the environmental performance of wastewater treatment systems across their life cycle due to their high energy use and infrastructure requirements.^6^ While these perspectives are often addressed separately, their integration is essential to avoid unintended trade-offs and identify best management options.

Quantitative microbial risk assessment (QMRA) provides a well-established framework to estimate infection risks from pathogenic microorganisms in the environment. By combining exposure assessment with pathogen-specific dose–response relationships, QMRA enables detailed evaluation of health risks for defined populations and exposure scenarios. Numerous studies have applied QMRA to wastewater-related exposures.^7–12^ Recreational water contact in surface waters has been consistently identified as an elevated infection risk.^12–14^ In addition to site-specific applications, QMRA is also widely used in regulatory and water quality management contexts to support generalized treatment targets and acceptable risk thresholds.

Within environmental assessments of wastewater systems, LCA provides a standardized framework to quantify the impacts of products and services across their life cycle.^15^ While LCA covers a wide range of impact categories, such as climate change, eutrophication, and toxicity, it currently lacks an impact category for pathogen-related human health effects.^16–18^ As a result, pathogen emissions from wastewater systems are not represented as elementary flows, and exposure-driven health impacts are omitted from LCIA. These gaps limit the ability of LCA to holistically evaluate wastewater treatment and stormwater management options and may lead to burden shifting when pathogen risks are reduced at the expense of other environmental impacts (e.g., Petit-Boix et al.^4^ and Aramaki et al.^19^), or vice versa. A central methodological challenge is therefore not only estimating pathogen risks but translating concentration- and scenario-based microbial risk estimates into generalized emission-based characterization factors compatible with LCIA.

The potential for combining concepts from QMRA and LCA has already been recognized in previous work;^17,18,20–23^ however, systematic integration of pathogen emissions from wastewater treatment plants (WWTPs) into LCA remains limited. A key point of convergence of LCA and QMRA is the use of disability-adjusted life years (DALY) as a common metric to express human health burdens. DALY represents the years of life lost and the years lived with disability attributable to disease. The aforementioned studies have conceptually applied this approach in the context of wastewater treatment, for example, by quantifying pathogen-related health burdens using QMRA alongside other human health impacts within LCA. Heimersson et al.^17^ reported that microbial risks make up 20 % of the human health impacts in the context of sewage sludge management while Bhatt et al.^20^ even report 86 % for a sewage treatment plant. While these studies underline the importance of pathogens and the necessity to integrate pathogens into LCIA, they largely focus on single sites and do not operationalize pathogen impacts as transferable characterization factors (CFs) compatible with regionalized LCIA frameworks. Thus, a national-scale or more general perspective remains lacking due to methodological and data-related gaps.

To address this gap, this study presents the first spatially explicit framework for deriving pathogen-related CFs for LCIA from wastewater emissions. By linking pathogen emissions from WWTPs to downstream human health outcomes through fate, exposure, dose–response, and health burden assessment, the framework translates pathogen emissions into regionalized CFs expressed as DALY per emitted pathogen. This provides a methodological basis for integrating pathogen-related human health impacts into LCIA and enables their quantification alongside established environmental impact categories.

The framework is designed in a modular manner to support future adaptation to different regions, pathogens, exposure pathways, and levels of data availability. Switzerland serves as a high-resolution proof-of-concept implementation because of the availability of validation data for the fate model. Six representative pathogens are included: *Escherichia coli (E. coli)*, *Campylobacter jejuni*, *Cryptosporidium parvum*, norovirus, adenovirus, and rotavirus.

## Method

### Pathogenic Emissions

We assess pathogen loads entering the aquatic environment from WWTPs, while other potential pathogen sources, such as agricultural runoff, wildlife, and combined sewer overflows, are excluded. Influent wastewater concentrations of six pathogens are assumed to be spatially and temporally constant and derived from literature values (Table 1). Where reported concentrations include both pathogenic and non-pathogenic organisms, they are multiplied by a literature-derived pathogenic fraction; concentrations already reported for a specific pathogen or in infectious units are used without further adjustment. For example, a pathogenic fraction of 0.08 (literature range: 0.07–0.09 ^24^) is applied to culturable *E. coli*, without differentiation among pathotypes. Treatment stages of each WWTP^25^ are mapped to the classification proposed by Tchobanoglous et al.,^26^ and pathogen-specific removal efficiencies are assigned based on the corresponding treatment configuration (Table S2). Effluent concentrations are then calculated as

1 $$C_{effluent,p}=C_{\mathrm{influent},p}\cdot \left(1-\eta_{p}\right)$$

**Table 1:** Infectivity-Adjusted Pathogen Concentrations in Wastewater Influent^24,53–55^ and Effluent Concentrations under Secondary and Advanced Wastewater Treatment Processes^a^

pathogen	influent concentration	effluent concentration (secondary removal)	effluent concentration (advanced removal)	unit
*E. coli*	4.00 × 10^8^	4.00 × 10^6^	5.02 × 10^3^	CFU L^–1^
*Campylobacter jejuni*	5.96 × 10^3^	5.96 × 10^1^	1.5 × 10^–1^	CFU L^–1^
Norovirus	5.01 × 10^4^	1.58 × 10^3^	1.99 × 10^1^	PFU L^–1^
Adenovirus	2.66 × 10^3^	8.41 × 10^1^	1.06	IU L^–1^
Rotavirus	4.25	1.34 × 10^–1^	1.69 × 10^–3^	PFU L^–1^
*Cryptosporidium parvum*	8.71 × 10^1^	4.90	4.36 × 10^–5^	oocysts L^–1^

a (CFU: colony-forming units (culture-based measure of viable bacteria); IU: infectious units; PFU: plaque-forming units (measure of infectious viral particles)).

where *C*_effluent,*p*_ is the concentration of pathogen *p* in the treated wastewater effluent (pathogens m^–3^), *C*_influent,*p*_ is the concentration of pathogen *p* in the influent wastewater before treatment (pathogens m^–3^), and *η*_*p*_ is the pathogen-specific removal efficiency of the wastewater treatment process (−).

Effluent concentrations are combined with WWTP-specific discharge volumes reported in the HydroWASTE database^25^ to estimate annual pathogen emissions entering receiving water bodies. Each WWTP discharge is assigned to the nearest river or lake segment, where effluents are assumed to mix instantaneously and completely within the receiving segment. The resulting mixed concentration serves as the starting concentration for downstream fate modeling.

### Fate

Pathogen concentrations are modeled within a segment-resolved river–lake network derived from HydroRIVERS, RiverATLAS, and HydroLAKES,^25,27–30^ representing surface waters under steady-state conditions. Concentrations are determined by the combined effects of hydrological transport, dilution, and pathogen decay.

In rivers, pathogen concentrations are routed downstream along connected segments, assuming advective transport, complete lateral mixing within each segment, and first-order decay. Segment-specific travel times are derived from the mean discharge and segment length. Concentrations are updated sequentially along the river network by accounting for upstream inputs, transport, dilution, and decay processes.

In lakes, pathogen concentrations are calculated within the well-mixed surface mixed layer following the method proposed by Wells and Troy^31^ and using a steady-state mass balance that accounts for inflows, outflows, and pathogen decay. To account for elevated pathogen concentrations near river inflows, localized concentrations within lake plume zones were calculated separately. These plume zone concentrations complement the mixed-layer concentrations and allow the representation of spatial concentration gradients within lakes.

Pathogen decay is represented by two first-order inactivation processes. Temperature-dependent inactivation is modeled using an Arrhenius-type formulation based on monthly mean summer water temperatures (June–August) from station-based observations provided by the Swiss Federal Office for the Environment. Ultraviolet radiation (UV)-driven decay rates are calculated from satellite-derived UV irradiance^32^ and site-specific light attenuation coefficients estimated from Secchi depth observations, which are used as a proxy for water turbidity.^33–36^ For lakes, Secchi depth observations are obtained from the Datalakes database.^37^

The combined effects of transport, dilution, and pathogen decay determine pathogen concentrations at designated bathing sites according to eq 2:

2 $$C_{\mathrm{bathingsite},p}=C_{\mathrm{mixed},p}\exp\left(-k_{\mathrm{UV},p}\tau_{\mathrm{lake}}-k_{\mathrm{temperature},p}t\right)$$

where *C*_bathing site,*p*_ is the concentration of pathogen *p* at the bathing site (pathogens m^–3^), *C*_mixed,*p*_ is the pathogen concentration after mixing within the receiving water body (pathogens m^–3^), *k*_UV,*p*_ is the pathogen-specific first-order photo-inactivation rate constant (d^–1^), *τ*_lake_ is the residence time within the lake photic zone (d), *k*_temperature,*p*_ is the pathogen-specific first-order temperature-dependent decay rate constant (d^–1^), and *t* is the pathogen travel time from the emission source to the bathing site (d).

The resulting pathogen concentrations at bathing sites serve as inputs to the exposure assessment.

### Exposure

Exposure is defined as contact between the population and surface waters at designated bathing sites. Site-specific pathogen concentrations *C*_bathingsite,*p*_ are combined with population distribution and recreational water-use behavior to estimate pathogen exposure at the reported bathing locations (eq 3):

3 $$D_{i,p,a}=C_{\mathrm{bathingsite},i,p}\cdot V_{\mathrm{ing},a}\cdot n_{\exp,a}$$

where *D*_*i*,*p*,*a*_ is the annual ingested dose of pathogen *p* for an individual in age group *a* assigned to bathing site *i* (pathogens person^–1^ yr^–1^), *C*_bathing site,*i*,*p*_ is the modeled concentration of pathogen *p* at bathing site *i* (pathogens m^–3^), *V*_ing,*a*_ is the accidentally ingested water volume per recreational water-contact event for age group *a* (m^3^ event^–1^), and *n*_exp,*a*_ is the annual number of recreational water-contact events for age group *a* during the bathing season (events person^–1^ yr^–1^).

Gridded Swiss population density (1 km × 1 km) by age band is assigned to the nearest bathing site reported in the European database^38,39^ based on Euclidean distance, assuming that recreational activities predominantly occur at the closest accessible location.

Recreational water-use behavior is represented through probabilistic descriptions of the visit frequency and accidental water ingestion per visit. These age- and sex-specific distributions are based on a Dutch survey, which provides empirical data on swimming frequency, ingestion volumes, and the proportion of the population engaging in recreational water activities.^11^ While these data do not originate from Switzerland, they are used as proxies for Western European behavior in the absence of country-specific behavioral studies.

For each bathing site, individual pathogen doses per visit are calculated as the product of modeled local pathogen concentration and ingested water volume during one recreational event with the reported anual frequency (eq 3). Annual individual doses are then obtained by combining per-visit doses with the annual frequency of recreational exposure assumed to occur between June and August and reported by Schets et al.^11^ These doses form the basis for the subsequent dose–response assessment.

### Dose–Response

Pathogen-specific dose–response models are used to estimate the probability of infection or illness as a function of the individual pathogen dose at each bathing site (Table S12), following literature recommendations for model selection and parameterization.^12,40–43^ Depending on the pathogen, either β-Poisson or hypergeometric dose–response models are applied.

The probability of illness is calculated as

4 $$P_{\mathrm{ill},i,p,a}=f_{p}\left(D_{i,p,a}\right)\cdot r_{\mathrm{ill}|\inf,p}$$

where *P*_ill,*i*,*p*,*a*_ is the probability of illness for pathogen *p*, age group *a*, and bathing site *i*, *f*_*p*_(*D*_*i*,*p*,*a*_) is the pathogen-specific dose–response function evaluated at the annual individual dose *D*_*i*,*p*,*a*_, and *r*_ill|inf,*p*_ is the illness-to-infection ratio applied when the underlying dose–response model differ in their reported health end points. For dose–response models that directly estimate illness, *r*_ill|inf,*p*_ is equal to 1. The illness-to-infection ratio is assumed constant across age bands.

Each recreational water contact is treated as an independent exposure event, and infections caused by different pathogens are assumed to be independent. Secondary transmission is not considered. Due to the lack of age- and sex-specific dose–response relationships, a single dose–response curve is applied to the affected population for each pathogen. The modular structure of the framework allows the incorporation of alternative dose–response models and pathogen-specific parameterizations as additional data become available.

### Health Burden Assessment

Human health impacts are quantified as annual DALY at the end-point level. Annual illness cases are converted into health burden according to eq 5:

5 $$\mathrm{DAL}Y_{i,p,a}=\mathrm{Il}\ln\mathrm{esse}s_{i,p,a}\cdot \mathrm{DAL}Y_{\mathrm{percase},p}$$

where *DALY*_*i*,*p*,*a*_ is the annual health burden attributable to pathogen *p*, age group *a*, and bathing site *i*, *illnesses*_*i*,*p*,*a*_ is the corresponding annual number of illness cases, and *DALY*_per case,*p*_ is the pathogen-specific DALY associated with a single illness case.

Since global health burden estimates from the Global Burden of Disease (GBD) are reported as annual DALY per 100,000 population,^44^ pathogen-specific DALY/case values are derived by combining these estimates with annual incidence rates (illnesses per 100,000 population) collected from the literature. Because of limited availability of incidence data, European, German, and Swiss average values are used for the assessed pathogens. For adenovirus, laboratory-confirmed cases^45^ are multiplied by a factor of 100 to account for underreporting.

### Characterization Factor Derivation

The derived health burdens are related to pathogen emissions to obtain CFs suitable for the LCIA. CFs are calculated separately for each pathogen and river basin as

6 $$CF_{b,p}=\frac{\mathrm{DAL}Y_{b,p}}{E_{b,p}}$$

where *CF*_*b*,*p*_ is the CF for pathogen *p* in basin *b* (DALY per emitted pathogen), *DALY*_*b*,*p*_ is the total annual health burden attributable to pathogen *p* within basin *b*, and *E*_*b*,*p*_ is the corresponding annual pathogen emission.

CFs are calculated at both the national level and for the four major Swiss drainage basins (Rhine, Rhône, Po, and Danube) based on the HydroBASINS delineation.^28^

### Model Validation

Measured pathogen concentration data provided by the cantons of Zurich, Thurgau, and Ticino were used to evaluate the performance of the fate component of the model. Available observations are compared to modeled pathogen concentrations at the corresponding river and lake segments downstream of WWTP discharges. Because of limited data availability and the static model setup, validation focuses on order-of-magnitude agreement rather than exact concentration matches. Temporal variability is not explicitly validated as the model operates on monthly mean environmental conditions.

### Uncertainty and Global Sensitivity Analysis

Uncertainty and global sensitivity analysis (GSA) are conducted to evaluate how uncertain model inputs influence the estimated health burden. Uncertain parameters in the fate and transport, exposure, infection, dose–response, and health impact components are assigned probability distributions based on literature values, empirical ranges, or model assumptions, following the guidelines by Burmaster and Anderson^46^ and common practice in LCA.

Uncertainty is propagated using Monte Carlo simulations (*n* = 10′000). The output is total annual DALY, obtained by summing pathogen-specific estimates across all Swiss locations. A variance-based Sobol GSA is then applied to identify the main drivers of output variability.^47,48^ For the Sobol analysis, 512 base samples are generated across the uncertain parameter space and transformed according to the specified distributions, resulting in 19’456 model evaluations.

To further test the nonlinearity of the model, effluent pathogen concentrations are scaled uniformly across all WWTPs while other parameters are held constant. This is done to specifically test the nonlinearity of the model. Scaling factors range from the baseline scenario (1×) to extreme stress scenarios up to × 10^8^, allowing assessment of the framework’s dynamic range and numerical stability. Higher factors are included to explore the dynamic range and numerical stability of the proposed fate–exposure–effect framework.

## Results and Discussion

### Wastewater Treatment Plants

Figure 1A presents the spatial distribution of WWTPs (N = 759) in Switzerland, including their design capacity and treatment level. Treatment levels are classified as “Secondary” or “Advanced” for the case of Switzerland according to the HydroWASTE database.^25^ Based on Tchobanoglous et al.,^26^ secondary treatment is defined as biological treatment processes for organic matter and nutrient removal in our study. In Switzerland, advanced treatment includes additional steps such as ozonation and sand filtration,^49^ with the primary purpose of removing micropollutants (MP), which also result in higher overall pathogen removal efficiencies.^50,51^ The concentrations of the pathogens in wastewater influent and effluent resulting from both Secondary and Advanced treatment can be found in Table 1. The spatial distribution of WWTPs closely mirrors Switzerland’s settlement structure, with a high density of facilities in urbanized lowland regions (i.e., Swiss Plateau). The total capacity of population equivalent served amounts to 11.4 × 10^6^, corresponding to an annual treated effluent volume of 1.9 × 10^9^ m^3^ yr^–1^. Approximately two thirds of the WWTPs are equipped with advanced treatment, while one third operates at the secondary treatment level. Regional differences in treatment level are visible, with a higher proportion of secondary treatment plants in southern Switzerland (i.e., Ticino) compared to the Swiss Plateau, which hosts predominantly advanced treatment WWTPs. Importantly, the level of detail in the database is relatively coarse and might not represent any additional treatment stages installed, such as those related to MP removal.^52^ With additional treatment stages, the effluent concentrations might be lower than the ones reported in Table 1.

**Figure 1:**
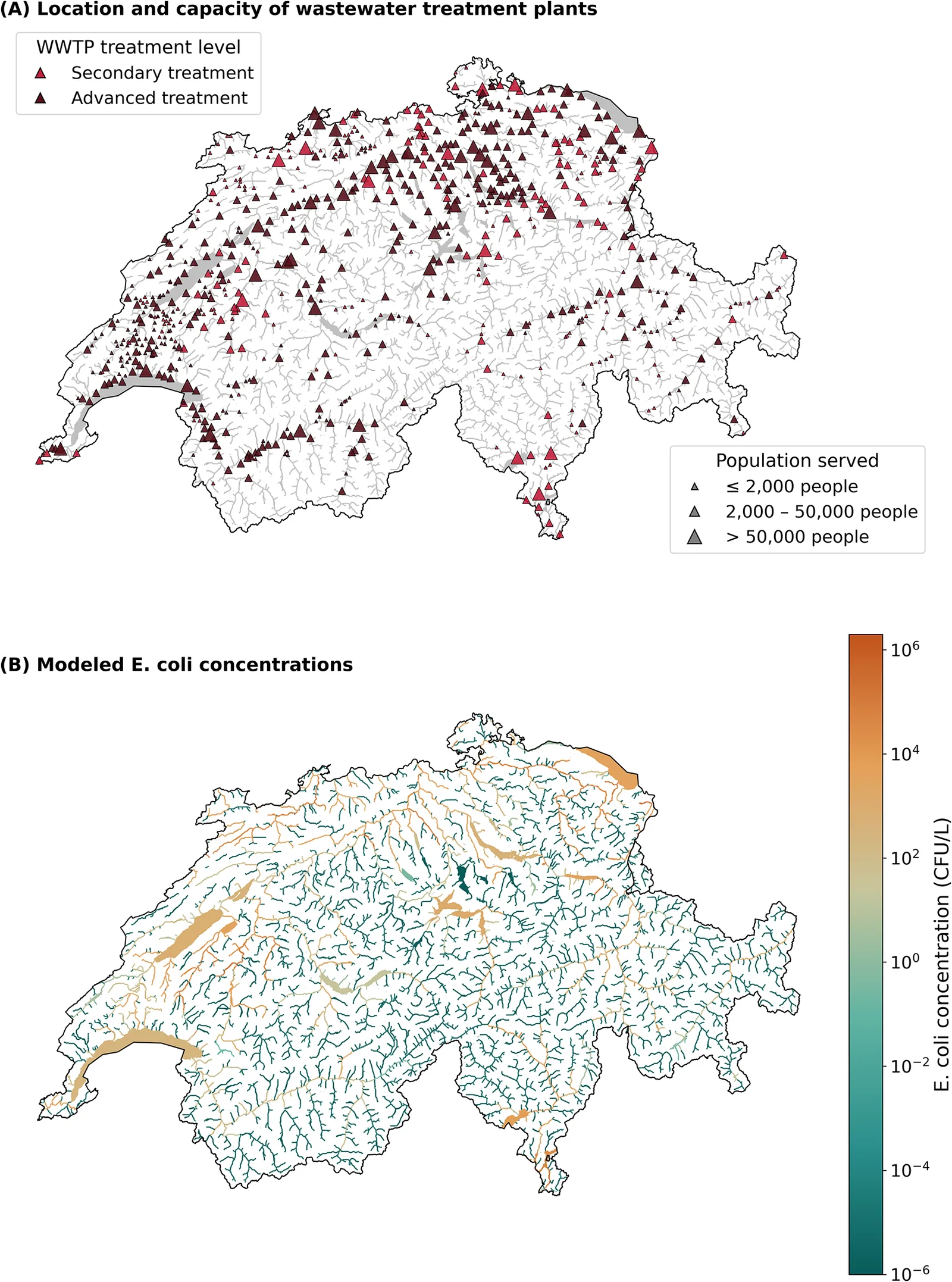
(A) Location, treatment level, and capacity (population equivalent served) of wastewater treatment plants in Switzerland.^25^ (B) *E. coli* concentrations (CFU L^–1^) in Swiss rivers and lakes; *E. coli* is used as an indicator for the other pathogens as the gradients look similar. Lake-specific variability is aggregated due to the high spatial resolution required for individual lake representation. Zero values are assigned to 10^–6^ CFU L^–1^.

### Fate

We cover in total 7,899 river segments and 882 lakes defined by the RiverATLAS.^30^ The river network represents a total modeled length of 13,321 km, with segment lengths typically ranging from 0.10 to 4.73 km (5th–95th percentile) (Table S6). The lakes cover a cumulative area of 1,388 km^2^, with areas ranging from 0.011 to 1.40 km^2^ between the 5th and 95th percentiles (Table S7).

Modeled concentrations for *E. coli*, as a representative pathogen in Figure 1B, span several orders of magnitude (1.2 × 10^–3^ to 1.5 × 10^6^ CFU L^–1^) across the Swiss surface water network, indicating pronounced spatial heterogeneity. Similar spatial patterns are observed for all six pathogens in Figures S2–S6, with differences between pathogens driven by variations in wastewater influent concentrations, pathogen-specific treatment efficiencies and pathogen-specific decay parameters (Table S4).

Overall, most river and lake segments exhibit very low pathogen concentrations, while a limited number of locations show substantially elevated values, resulting in distinct hot spots. Nonzero concentrations occur more frequently in lakes, whereas rivers display stronger short-range variability and higher local maxima.

In rivers, elevated concentrations typically occur in short downstream stretches immediately following wastewater discharge. These local peaks arise because effluents enter relatively small receiving water volumes and are transported downstream before substantial dilution or decay occurs. For example, the highest pathogen concentrations are observed in the Fribourg area, resulting from the combination of high discharge volumes, secondary treatment levels, and a limited dilution capacity in receiving rivers. Although larger rivers provide greater dilution capacity, the high density of wastewater discharges results in elevated cumulative pathogen loads in the Swiss Plateau (e.g., Aare and Limmat), leading to higher concentrations compared to those in sparsely populated alpine regions with a lower number of WWTPs.

In contrast, lakes generally exhibit lower but more spatially widespread concentrations. Incoming pathogen loads from upstream rivers and wastewater discharges are distributed over much larger water volumes and mixed over longer time scales. This results in smoother spatial concentration patterns, with fewer extreme peaks but a greater number of lake segments showing detectable pathogen levels. Among the lakes, Lake Zurich and Lago Maggiore exhibit higher pathogen concentrations than other lakes (e.g., Lac Léman, Lago Lugano). When comparing modeled and measured data, our *E. coli* concentrations tend to lie at the upper end of the reported measurement range, which spans six orders of magnitude (Figures S9–S10). The overestimation of pathogen concentrations in Lake Zurich might be attributed to the limited resolution in the treatment data: HydroWASTE distinguishes only secondary and advanced treatment,^25^ so we assigned a single representative removal efficiency to each class. The WWTPs in the Lake Zurich catchment may achieve removal rates higher than our ‘advanced’ assumption through additional polishing/disinfection,^52,56^ leading to overestimated effluent loads. However, the modeled concentrations still lie within the range of the observed pathogen concentrations at Lake Zurich.

Overall, the results demonstrate that the translation of wastewater emissions into environmental concentrations is governed by the interaction between emission intensity, treatment efficiency, and hydrological structure. To illustrate the influence of wastewater treatment on environmental pathogen concentrations, we simulate four national treatment scenarios for *E. coli*: no treatment, primary, secondary, and advanced treatment. The results show a strong reduction in bathing-site concentrations with increasing treatment level (Figure S16). For *E. coli*, median bathing-site concentrations decrease from 8.8 × 10^4^ CFU L^–1^ under a no-treatment scenario to 1.1 CFU L^–1^ with advanced treatment, corresponding to a reduction of five orders of magnitude. This indicates that wastewater treatment efficiency is a dominant control on pathogen concentrations at recreational exposure locations. In addition, hydrology acts as a regulating mechanism that can either amplify local concentration peaks in small receiving rivers or attenuate emissions through dilution and mixing in large lakes. Consequently, emission magnitude alone is insufficient to predict downstream concentration patterns.

Given that Switzerland exhibits among the highest wastewater treatment efficiencies reported in the HydroWASTE database,^25^ the modeled concentrations represent a comparatively low-emission baseline relative to many global regions. In countries or regions with lower treatment coverage or efficiency, substantially higher environmental concentrations would be expected under similar hydrological conditions.

### Exposure and Health Impacts

The exposure assessment covers 503 recreational bathing sites in Switzerland, the majority of which are located at lakes. The fate modeling shows median *E. coli* exposure concentrations are higher at river bathing sites (6.7 × 10^3^ CFU L^–1^) than at lake bathing sites (3.1 × 10^2^ CFU L^–1^). Exposure concentrations span several orders of magnitude, ranging from 11.8 CFU L^–1^ (5th percentile) to 6.3 × 10^4^ CFU L^–1^ (95th percentile) for rivers, and from 1.8 CFU L^–1^ to 2.5 × 10^4^ CFU L^–1^ for lakes. Modeled concentrations are evaluated against monitoring data at bathing sites (Figures S8–S10).

Switzerland has a total population of approximately 8.3 × 10^6^ inhabitants in 2020,^38^ of which a modeled 5.2 × 10^6^ are exposed to waterborne pathogens through recreational activities in surface waters based on the behavioral study by Schets et al.^11^ Our study estimates a total annual human health burden of 1.15 × 10^3^ DALY attributable to exposure of the six pathogens in Switzerland (Figure 2). Of the 503 bathing sites, 67 % contribute nonzero health impacts. Nonzero health burden sites exhibit site-level impacts between 4.3 × 10^–7^ and 3.2 × 10^2^ DALY yr^–1^, while a small number of high-impact locations dominate the overall burden (median: 2.4 × 10^–3^ DALY yr^–1^; mean: 3.4 DALY yr^–1^). The ten sites with the highest impacts account for 64 % of total DALYs, and the top 50 sites contribute more than 95 % of the national total. This concentration of impacts indicates that human health impacts are strongly controlled by local combinations of pathogen load, hydrological conditions, and exposed population, rather than by emission volumes alone. The results demonstrate that emission magnitude alone is not predictive of downstream health risk; hydrological context determines exposure relevance.

**Figure 2:**
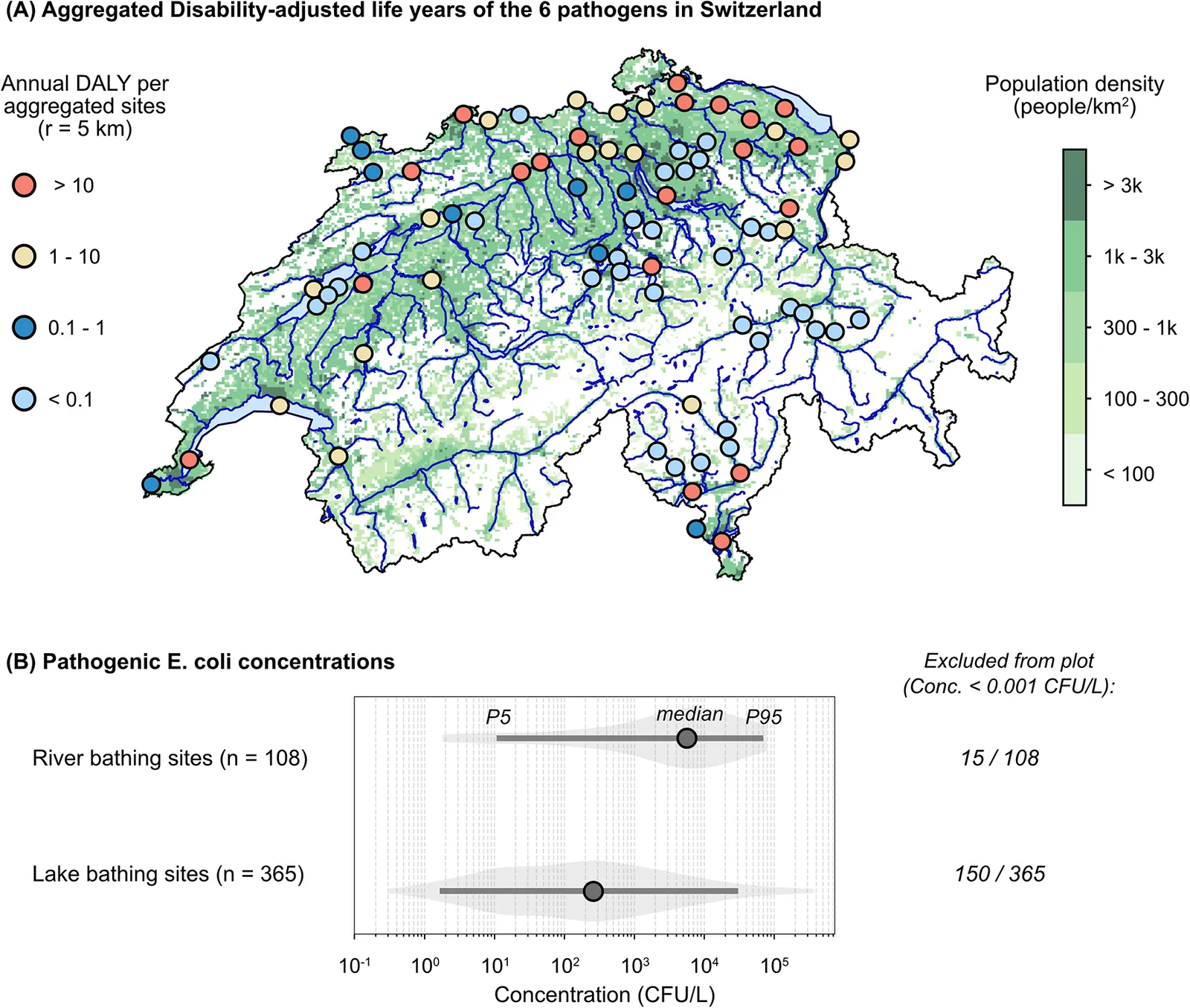
(A) Spatial distribution of pathogen-related health burden and (B) exposure concentrations in Switzerland. *E. coli* is used as a representative of the six pathogens in (B).

At the national level, *E. coli* contributes 983 DALY yr^–1^ (86 % of the total), followed by *Cryptosporidium parvum* (107 DALY yr^–1^; 9.3 %), Adenovirus (2.4 DALY yr^–1^; 0.2 %), and *Campylobacter jejuni* (46 DALY yr^–1^; 4.0 %). Norovirus (1.7 DALY yr^–1^; 0.1 %) and Rotavirus (7.7 DALY yr^–1^; 0.7 %) contribute marginally. The dominance of *E. coli* and *Cryptosporidium parvum* reflects the substantially higher modeled post-treatment effluent concentrations relative to viruses under the assumed secondary and advanced removal efficiencies (Table 1). Although viruses typically require lower doses to cause infection, their lower modeled effluent concentrations reduce their contribution to overall health impacts. These effects propagate through the exposure and dose–response assessment and ultimately determine the national health burdens. Additionally, the *E. coli* result provides an initial estimate based on the currently available data, using the EPEC O127 dose–response model as a proxy. Future pathotype-specific concentration and dose–response data would allow this estimate to be further refined.

To illustrate the role of wastewater treatment in shaping these health outcomes, additional treatment-stage scenarios are evaluated. In the absence of wastewater treatment, the annual health burden increases to approximately 1.0 × 10^6^ DALY yr^–1^. Introducing primary treatment reduces this burden to 4.4 × 10^5^ DALY yr^–1^, while secondary treatment further lowers it to 1.9 × 10^4^ DALY yr^–1^. Under advanced treatment, the estimated burden declines to only 32.8 DALY yr^–1^. Overall, this corresponds to a reduction of more than four orders of magnitude between the no-treatment and advanced-treatment scenarios.

When normalized to the total population, the estimated burden corresponds to approximately 1.37 × 10^–4^ DALY per person yr^–1^. This value is approximately 137 times higher than the tolerable burden of 10^–6^ DALY per person yr^–1^ used in the World Health Organization^57^ drinking-water guidelines. While this WHO reference level was developed for drinking-water exposures rather than recreational bathing, it provides useful context for the magnitude of the estimated burden. The value is also small compared to the total infectious disease burden in Switzerland (2.933 × 10^–2^ DALY per person yr^–1^ ^58^). Due to the high WWTP treatment efficiency (secondary and advanced) in Switzerland, the human health burdens from wastewater pathogen emissions are expected to be small compared to infectious diseases from other sources.

#### Characterization Factors

Based on the resulting health burdens, we derive basin-specific CFs at the HydroBASINS level (=4).^28^ The exposure assessment is restricted to the Swiss population; therefore, the CFs reflect health impacts occurring within Switzerland. Consequently, the CFs represent Swiss-attributable impacts per pathogen emitted within each basin rather than the full downstream impact across the entire hydrological catchment.

The resulting CFs show substantial variability across pathogens and basins (Figure 3). Mean basin-level CFs differ by up to five orders of magnitude between pathogens. The highest average CFs are observed for Rotavirus (mean: 4.7 × 10^–11^ DALY per emitted pathogen), followed by *Cryptosporidium parvum* (mean: 2.3 × 10^–11^), *Campylobacter jejuni* (mean: 7.4 × 10^–13^), Norovirus (mean: 3.6 × 10^–13^), and Adenovirus (mean: 1.6 × 10^–14^). In contrast, the bacterial indicator *E. coli* exhibits a markedly lower mean CF of 2.3 × 10^–16^ DALY per emitted pathogen, approximately five orders of magnitude below Rotavirus.

**Figure 3:**
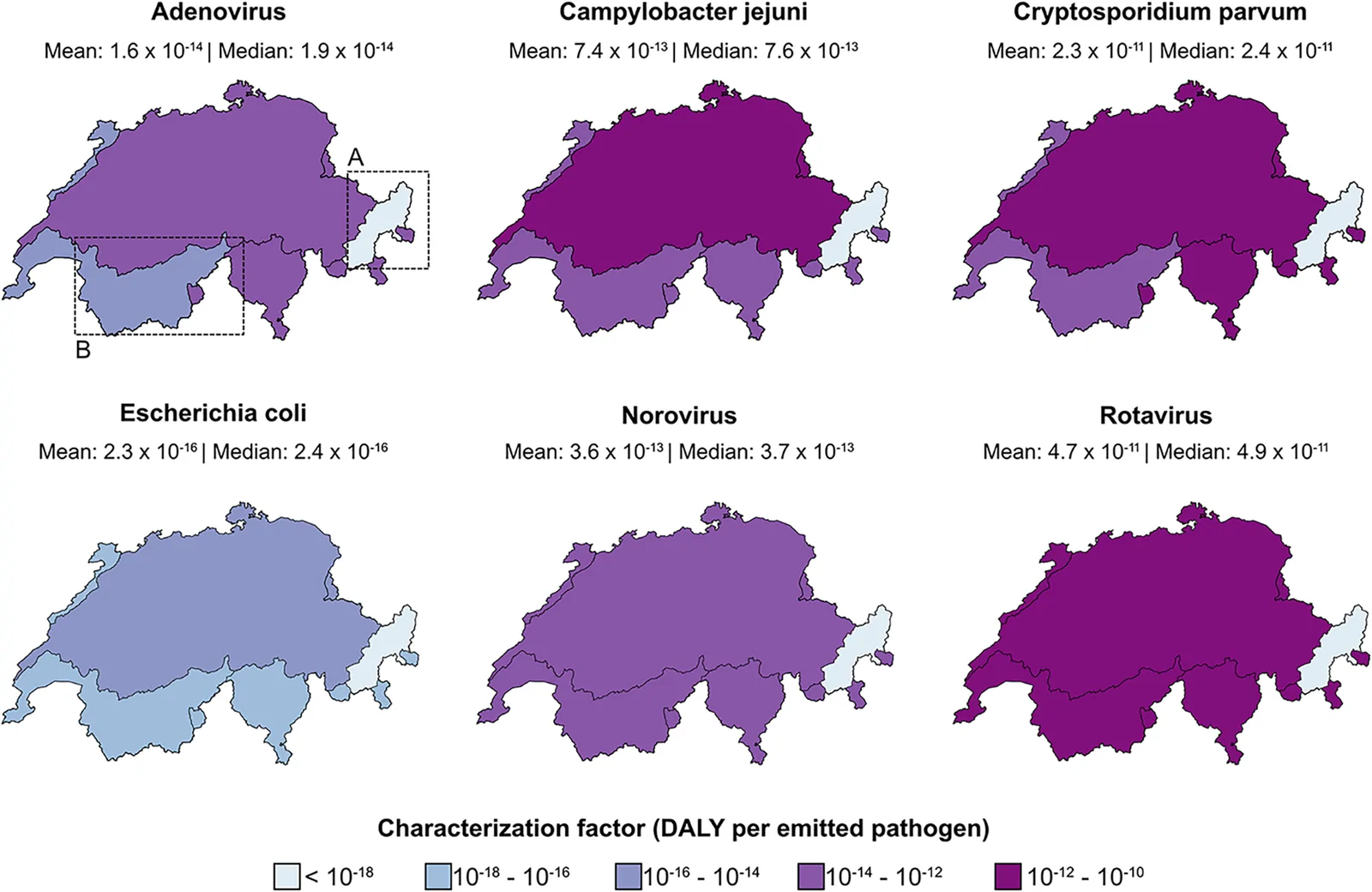
Basin-specific characterization factors for the six pathogens in Switzerland. The characterization factors are presented in DALY/emitted pathogen. Mean and median characterization factors are expressed as DALY per emitted pathogen (#) and are calculated at the HydroBASINS level, differentiating Rhine, Rhône, Po, and Danube.

These differences arise from pathogen-specific dose–response characteristics, illness probabilities, and DALY per illness case derived from GBD data Naghavi et al.^44^ The results therefore demonstrate that pathogen identity is a dominant determinant of health damage per emitted organism, and that the use of generic microbial indicators as proxies for pathogen-related impacts would lead to significant misrepresentation in LCA.

Regarding spatial variability, CFs emerge from the interaction of (i) population distribution, (ii) the spatial configuration of recreational bathing sites, and (iii) the number and location of WWTPs discharging into the surface water network. For example, regions such as Engadine and Rhône valley (boxes A and B in Figure 3) exhibit the lowest CFs as there is only one bathing site on the Rhône reported and none in Engadine. Specifically in the Rhône valley, there are many WWTPs reported but the dilution volume is also high, leading to relatively low pathogen concentrations in the river. This stands in contrast to regions on the Swiss Plateau (e.g., Aare and Limmat) where many bathing sites are reported and therefore, health risks occur, leading to high CFs. These findings highlight the necessity of combining hydrological fate modeling with spatially resolved exposure assessment when deriving pathogen-related CFs.

Because the resulting CFs are expressed in DALY per emitted organism, pathogen emissions from wastewater infrastructure can be directly compared with other human health impact categories at the end-point level. These CFs enable a direct comparison of wastewater infrastructure improvements with other health-relevant interventions within LCA, such as reductions in air pollution or toxic emissions. This creates a consistent end-point-level basis for evaluating sanitation investments alongside other environmental mitigation strategies.

### Uncertainty and Global Sensitivity Analysis

Uncertainty in the modeled national DALY burden is assessed using a Monte Carlo analysis, and the contribution of individual uncertain parameters to output variability is evaluated using a variance-based Sobol sensitivity analysis. The Monte Carlo results show that most simulations produce comparatively low total annual DALYs, whereas a small fraction of parameter combinations leads to substantially higher estimates (Figure S13). The median burden is 854 DALYs per year, with a 5th–95th percentile range of 46–9300 DALYs per year.

Sobol analysis is used to identify which uncertain input parameters contribute most to the variance in total DALYs. First-order indices (*S*_1_) describe the direct contribution of a parameter alone, whereas total-order indices (*S*_*T*_) include both direct effects and interactions with other uncertain parameters. The ranking is therefore interpreted primarily using *S*_*T*_, while differences between *S*_*T*_ and *S*_1_ are used as an indication of the interaction effects.

The highest total-order Sobol indices are obtained for the *E. coli* incidence scale (*S*_*T*_ = 0.70), the *E. coli* dose–response parameter *N*_50_ (*S*_*T*_ = 0.34), the uptake scale (*S*_*T*_ = 0.22), the *E. coli* dose–response parameter *α* (*S*_*T*_ = 0.19), and recreational exposure frequency (*S*_*T*_ = 0.16) (Figures S14–S15). Additional relevant contributions are associated with the affected population fraction (*S*_*T*_ = 0.095), the *E. coli* illness-per-infection parameter (*S*_*T*_ = 0.088), the DALY-per-case multiplier (*S*_*T*_ = 0.056), influent pathogen concentration (*S*_*T*_ = 0.041), and the *Cryptosporidium parvum* dose–response parameter *α* (*S*_*T*_ = 0.020). In contrast, temperature-dependent decay has a comparatively small total-order index (*S*_*T*_ = 0.0039). The substantial differences between *S*_*T*_ and *S*_1_ for the *E. coli* incidence scale (*S*_*T*_ – *S*_1_ = 0.54), *E. coli N*_50_ (*S*_*T*_ – *S*_1_ = 0.31), uptake scale (*S*_*T*_ – *S*_1_ = 0.18), and *E. coli α* (*S*_*T*_ – *S*_1_ = 0.17) indicate that their influence is driven strongly by interactions with other uncertain parameters. When the total-order indices are aggregated by model component and normalized by their sum across all parameters, effect-related parameters account for the largest share (72.5 %), followed by exposure-related parameters (24.8 %) and fate-related parameters (2.7 %). This indicates that uncertainty in incidence scaling, dose–response relationships, illness probabilities, and disease-burden assumptions has a substantially greater influence on output variability than uncertainty in environmental fate processes.

Overall, the uncertainty and sensitivity analyses show that the final DALY estimates are shaped primarily by epidemiological and health-effect uncertainty, particularly the *E. coli* incidence and dose–response assumptions. Exposure behavior remains relevant through uptake, recreational exposure frequency, and the affected population fraction, whereas fate-related uncertainty makes a comparatively small contribution in the present analysis. Future work should therefore prioritize refinement of the *E. coli* incidence scale, *N*_50_, *α*, and illness-per-infection assumptions, together with the principal exposure parameters. Influent pathogen concentrations represent the most influential fate-related input, while parameters governing treatment efficiency, temperature-dependent decay, discharge, mixing, and ultraviolet decay appear to be lower priorities for reducing uncertainty in total DALYs.

### Limitations

The results of this study should be interpreted in light of several modeling assumptions, data limitations, and scope choices. Effluent emissions from WWTPs are calculated from assumed spatially uniform influent concentrations and treatment efficiencies estimated based on the population served by each WWTP.^25^ The model therefore does not capture plant-specific removal efficiencies or temporal fluctuations in the treatment performance. Nationally representative measurements of pathogen concentrations in wastewater influent or effluent, ideally resolved by plant or region, would improve the spatial and temporal specificity of the emission estimates. Additional pathogen emission pathways, such as agricultural runoff or combined sewer overflows, are not included in the present modeling framework, although they are recognized as important contributors to pathogen emissions in surface waters.^14^ This boundary choice is consistent with the emission-based logic of LCA, which attributes impacts to specific inventoried sources. However, excluding these diffuse and episodic inputs may lead to an underestimation of the absolute pathogen loads in receiving waters. The modular structure of the proposed framework nevertheless allows for future integration of additional emission sources if corresponding inventory data become available.

Environmental fate modeling is based on long-term average conditions and does not fully resolve temporal variability in river discharge, water temperature, or event-driven hydrological conditions, such as heavy rainfall and urban runoff, which may influence pathogen transport and decay.^4^ The fate model considers the effects of water temperature and UV radiation on pathogen decay, while other hydrodynamic and particle-associated processes are not represented. Bacteria and viruses can attach to particles in aquatic systems, in which case sediments may act as a sink for deposited pathogens and pathogen decay may be slower due to reduced sunlight exposure.^59^ These dynamics may become particularly relevant during storm events, when high flows can cause sediment resuspension and short-term peaks in pathogen concentrations.^59^

The assessment is limited to six pathogens for which sufficient concentration, fate, and dose–response information is available, and therefore, does not capture the diverse sources of health impact from exposure to wastewater. The exposure assessment focuses exclusively on recreational water use and applies behavioral parameters from published QMRA studies conducted outside Switzerland, which may not fully reflect country-specific exposure patterns. This is particularly relevant because exposure behavior is a major driver of modeled health burdens. Expanding the assessment to additional exposure pathways would require spatially resolved information on exposure locations, exposed population groups, and exposure frequencies. Dose–response models used in this work are age-independent and are largely calibrated at higher pathogen doses, introducing uncertainty when extrapolated to environmentally relevant low-dose conditions. Health burden calculations rely on constant DALY factors across population groups and assume independence among pathogens when aggregating risks, thereby neglecting potential co-infections, differential susceptibility, secondary transmission within households, and antibiotic resistance dynamics. In this study, per-case DALY estimates are derived from Naghavi et al.,^44^ which are themselves model-based and not fully transparent at the per-case level. Future applications should assess the sensitivity of results to alternative per-case DALY estimates, including WHO-derived values or empirical estimates where available. These limitations reflect broader challenges in applying QMRA-based health-effect modeling within LCA.

Finally, the framework is applied to Switzerland, where environmental data governance is decentralized across the cantons. This limits the availability of harmonized national datasets on wastewater treatment, hydrology, water temperature, and related parameters. Because data collection and reporting practices vary across cantons, assembling a nationally representative dataset requires direct coordination with each cantonal authority, posing a major constraint on data acquisition.

## Implications for Life Cycle Assessment

Pathogen emissions represent a human health impact pathway that is largely absent from conventional LCA despite their relevance for wastewater, stormwater, and sanitation systems. By combining pathogen fate modeling, recreational exposure assessment, and health burden characterization, this study provides the first pathogen-specific CFs for emissions to rivers and lakes. The resulting framework establishes a direct link between microbial emissions and damage to human health, thereby extending LCIA beyond chemical toxicity and other established health-related impact categories.

The proposed CFs make pathogen-related health impacts operational within the emission-based LCA. They allow pathogen emissions from inventoried sources, such as WWTP effluents or other wastewater-related discharges, to be translated into comparable human health damage. The baseline inventory of pathogen loads derived from HydroWASTE^25^ illustrates how pathogen emissions can be represented as flows in life cycle inventory (LCI) modeling. This creates the basis for evaluating trade-offs in wastewater and stormwater management that are currently difficult to represent in LCA, such as the balance among energy use, chemical use, nutrient removal, greenhouse gas emissions, and microbial risk reduction. For example, decentralized wastewater treatment systems represent an important application area for future LCA studies, as their pathogen-related health impacts will depend on treatment performance and potential failure, discharge pathways, and proximity to exposed populations.

A central implication of the results is that pathogen characterization requires a regionalized assessment. Health impacts depend not only on emitted pathogen loads but also on environmental persistence, hydrological dilution, population distribution, and exposure opportunities. Generic or spatially averaged CFs would therefore risk obscuring important differences among the emission locations. The spatially explicit CFs developed here provide a way to account for these differences and identify locations where pathogen emissions are more likely to translate into substantial health burdens. The modeling framework itself can be transferred to other regions. Extending the approach to broader geographic contexts is particularly important for LCA, given the global nature of supply chains and the need for region-specific characterization, where sanitation conditions, exposure patterns, and pathogen emissions differ.

The framework also provides a basis for characterizing pathogen emissions under changing wastewater and stormwater conditions. Extreme precipitation, system failures, and overflow events may become increasingly relevant under climate change, leading to episodic pathogen releases that are not represented in the current LCA practice. The CFs developed in this study offer a practical starting point for characterizing such emissions and comparing management options on a common human health basis.

Although this study focuses on microbial pathogens, the same need for improved characterization also applies to other wastewater-related stressors, including MP, such as pharmaceutical substances, per- and polyfluoroalkyl substances, or heavy metals.^60–62^ Their integration into LCA remains challenging because health-effect characterization is limited by missing dose–response relationships and by potential mixture or synergy effects among compounds.^63^ In addition, MP may affect not only human health but also freshwater, marine, and soil ecosystems.^63^ Addressing these stressors alongside pathogens would further strengthen the assessment of wastewater management options, but requires stressor-specific effect models beyond the scope of the present study.

By making pathogen-related health impacts spatially explicit, the proposed framework allows pathogen control to be represented as a quantifiable outcome of sanitation and water management in the LCA. Wastewater, stormwater, and sanitation systems can therefore be compared not only in terms of resource use and environmental emissions but also in terms of their contribution to reducing human exposure to waterborne pathogens. This brings LCA closer to representing both the environmental burdens and public health functions of these systems.

## Supplementary Material



## Notes

Model description, pathogen-specific concentration maps, model validation results, additional global sensitivity analysis results, limitations (PDF)
