# Supplementary material for: Spatially Explicit Assessment of Pathogen-Related Health Impacts in Life Cycle Assessment
Source: Environ Sci Technol. 2026 Sep 16;60(38):27192–203. doi: 10.1021/acs.est.6c10006 (PMC13629626; doi:10.1021/acs.est.6c10006)
Supplement: Supplementary file 1 [file es6c10006_si_001.pdf]

Supporting information

Spatially Explicit Assessment of Pathogen-Related Health Impacts in Life Cycle Assessment

Vanessa Schenker, Yao Zhou and Stephan Pfister

Swiss Federal Institute of Technology Zurich, Chair of Ecological Systems Design,

Laura-Hezner-Weg 7, CH-8093 Zurich

Appendix includes:

- Number of pages: 42
- Number of figures: 18
- Number of tables: 15

List of Figures

1 (A) Location, treatment level, and capacity (population equivalent served) of wastewater treatment plants in Switzerland[25]. (B) *E. coli* concentrations (CFU L^{-1}) in Swiss rivers and lakes; *E. coli* is used as an indicator for the other pathogens as the gradients look similar. Lake-specific variability is aggregated due to the high spatial resolution required for individual lake representation. Zero values are assigned to 10^{-6} CFU L^{-1}. 13

2 (A) Spatial distribution of pathogen-related health burden and (B) exposure concentrations in Switzerland. *E. coli* is used as a representative of the six pathogens. 19

3 Basin-specific characterization factors for the six pathogens in Switzerland. The characterization factors are presented in DALY/emitted pathogen. Mean and median characterization factors are expressed as DALY per emitted pathogen (#) and are calculated at the HydroBASINS level, differentiating Rhine, Rhône, Po and Danube. 21

A1 Flowchart of the algorithm for computing the site-specific pathogen concentration on a river segment. A18

A2 Modeled Adenovirus concentration expressed in Infectious Units per L. . . . A20

A3 Modeled Campylobacter jejuni concentration expressed in CFU per L. A21

A4 Modeled Cryptosporidium parvum concentration expressed in oocysts per L. A21

A5 Modeled Norovirus concentration expressed in PFU per L. A22

A6 Modeled Rotavirus concentration expressed in PFU per L. A22

A7 Spatially resolved comparison between measured *E. coli* concentration distributions and model predictions for selected river bathing sites. Each boxplot corresponds to one monitoring location: top and bottom of the box indicate the interquartile range (25th–75th percentile), with the horizontal line representing the median; lower and upper whiskers correspond to the minimum and maximum observed concentrations; sample sizes (n) are indicated by the number of individual measurement points contributing to each distribution; red points indicate site-specific model predictions. A24

A8 Spatially resolved comparison between measured *E. coli* concentration distributions and model predictions for selected lake bathing sites. Each boxplot corresponds to one monitoring location: top and bottom of the box indicate the interquartile range (25th–75th percentile), with the horizontal line representing the median; lower and upper whiskers correspond to the minimum and maximum observed concentrations; sample sizes (n) are indicated by the number of individual measurement points contributing to each distribution; red points indicate site-specific model predictions. A25

A9 Comparison between measured *E. coli* concentration distributions and model predictions aggregated by water body using the arithmetic mean of modeled concentrations. Boxplots summarise observed bathing-water measurements: top and bottom of the box indicate the interquartile range (25th–75th percentile), with the horizontal line representing the median; lower and upper whiskers correspond to the minimum and maximum observed concentrations; sample sizes (n) are indicated by the number of individual measurement points contributing to each distribution; red points indicate model predictions. . . . A25

A10 Comparison between measured *E. coli* concentration distributions and model predictions aggregated by water body using the median of modeled concentrations. Boxplots summarise observed bathing-water measurements: top and bottom of the box indicate the interquartile range (25th–75th percentile), with the horizontal line representing the median; lower and upper whiskers correspond to the minimum and maximum observed concentrations; sample sizes (n) are indicated by the number of individual measurement points contributing to each distribution; red points indicate model predictions. A26
A11 Frequency distribution for the aggregated population groups divided in females, males and kids. N = 10000 . . . . A27
A12 Water uptake distribution for the aggregated population groups divided in females, males and kids. N = 10000 . . . . A27
A13 Monte Carlo uncertainty distribution of total annual DALYs. The upper panel shows the kernel density estimate of total DALYs across 10,000 Monte Carlo simulations, with vertical lines indicating the 5th percentile, median, and 95th percentile. The lower panel shows the individual Monte Carlo realizations as jittered points. The x-axis is shown on a logarithmic scale to improve readability of the right-skewed distribution. A36
A14 Sobol sensitivity indices for the most influential uncertain parameters in the total annual DALY estimate. Bars show total-order indices (S_T), which include both direct effects and interactions, while black points show first-order indices (S_1). Colours indicate the model component to which each parameter belongs. A38
A15 Relative contribution of fate, exposure, and effect parameters to the summed total-order Sobol indices for total annual DALYs. A39

A16 Modeled *E. coli* concentrations at bathing sites across wastewater treatment scenarios. Points represent individual site-level concentrations. Boxes show the interquartile range (25th–75th percentile), the thick horizontal line indicates the median, and whiskers indicate the 5th and 95th percentiles. Concentrations are shown on a logarithmic scale in CFU L^{-1}. A40
A17 Pathogen-specific contributions to total annual DALYs across wastewater treatment scenarios. Stacked bars show the summed DALY burden attributable to each pathogen under each treatment scenario, with bar heights representing the total national annual health burden. A41
A18 Sensitivity of health impacts to pathogen concentrations. Effluent concentrations are scaled over eight orders of magnitude relative to the baseline scenario, and the resulting relative DALYs are shown as the median (points) and the 5–95 % uncertainty range (shaded area). The dashed line indicates linear scaling for reference. A42

List of Tables

1 Infectivity-adjusted pathogen concentrations in wastewater influent[24,53–55] and effluent concentrations under secondary and advanced wastewater treatment processes. (CFU: colony-forming units (culture-based measure of viable bacteria); IU: infectious units; PFU: plaque-forming units (measure of infectious viral particles)). 14

A1 Log removal ranges for selected pathogens by treatment process. A9

A2 Treatment efficiencies for different pathogens by treatment level A9

A3 Model input variables (described below) A10

A4 Pathogen-specific model parameters for temperature-dependent decay[64]. . . . A13

A5 UV inactivation rate constants (k_{H}) for selected pathogens. A16

A6 Summary statistics of river segment lengths derived from RiverATLAS. . . . A19

A7 Summary statistics of lake areas derived from RiverATLAS. A19

A8 Pathogen concentration statistics across Swiss rivers and lakes. Summary statistics exclude zero-concentration entries (n = 19,310 total locations). . . A19

A9 Pathogen concentration statistics for river bathing sites (Switzerland). Statistics exclude zero-concentration entries ($n = 108$ total rows). A20

A10 Pathogen concentration statistics for lake bathing sites (Switzerland). Statistics exclude zero-concentration entries ($n = 395$ total rows). A20

A11 Modeled concentration ranges at bathing sites in Switzerland compared with representative mean concentrations reported in the literature of rivers and lakes across Europe, North and South America. A26

A12 Dose–response models and parameters of pathogens assessed in this study. . A29

A13 Sensitivity parameters used in the Monte Carlo uncertainty analysis and Sobol global sensitivity analysis. A32

A14 Summary statistics of the Monte Carlo uncertainty analysis for total annual DALYs. A36

A15 Sobol sensitivity indices for all uncertain parameters. Parameters are ranked by the total-order Sobol index (S_T). A37

Pathogenic emissions

Wastewater Treatment

The initial pathogen concentration at the point of discharge (C_0) is computed using the pathogen concentration in raw wastewater (C_{raw}) and the pathogen removal efficiency (η) associated with the treatment level of each WWTP. Representative influent pathogen concentrations (C_{raw}) are obtained from literature sources and are summarized in Table 1.

Each WWTP is spatially assigned to the nearest river segment in the hydrological network, which is assumed to represent the receiving water body of the effluent discharge. The effluent is then introduced into the transport model at the corresponding river segment location.

Pathogen removal efficiencies are assigned based on the treatment level ($LEVEL$) reported in the HydroWASTE database, which distinguishes between "Primary", "Secondary", and "Advanced" treatment. Primary treatment represents physical removal of suspended solids through sedimentation processes[26]. Secondary treatment includes biological treatment processes such as activated sludge systems, where microorganisms degrade organic matter under aerobic conditions[26]. Advanced treatment represents additional polishing steps including filtration and disinfection processes that further reduce pathogen concentrations[26].

To derive representative removal efficiencies for each treatment level, log removal values reported in the literature for different treatment processes are compiled (Table A1). For a treatment process with an average log removal L, calculated as the geometric mean of the reported minimum and maximum values, the corresponding removal efficiency η is calculated as:

$$\eta(\%) = \left(1 - \frac{1}{10^L}\right) \times 100 \tag{7}$$

The overall removal efficiency for each treatment level is then derived by combining the representative removal efficiencies of the treatment processes included in that treatment

level. The resulting pathogen-specific removal efficiencies used in the model are summarized in Table A2.

The effluent pathogen concentration released into the receiving water body is calculated as:

$$C_0 = C_{\text{raw}} \cdot (1 - \eta) \tag{8}$$

Table A1: Log removal ranges for selected pathogens by treatment process.

**Treatment Process**	**Bacteria**		**Viruses**		**C. parvum**		**Ref.**
	Min	Max	Min	Max	Min	Max	
Primary treatment	0.0	1.0	0.0	1.0	0.5	1.0	[53]
Biological Nutrients Removal	1.0	2.0	0.0	2.0	0.0	1.0	[53]
Ozonation	2.0	2.0	2.0	2.0	2.0	2.0	[50]
Sand filtration	1.6	3.8	1.1	3.3	3.5	6.1	[51]

Note: Log removal values represent literature ranges for each treatment process.

Table A2: Treatment efficiencies for different pathogens by treatment level

**Pathogen**	**Primary (%)**	**Secondary (%)**	**Advanced (%)**
E. coli	68.38	99.00	99.999
Campylobacter jejuni	68.38	99.00	99.999
Norovirus	68.38	96.84	99.96
Adenovirus	68.38	96.84	99.96
Rotavirus	68.38	96.84	99.96
Cryptosporidium parvum	82.22	94.38	99.9999

Fate

Overview

A spatially explicit fate model is implemented in Python and is developed to estimate pathogen concentrations at bathing sites. Bathing sites are assigned to the nearest river segment in the hydrological network. Pathogen transport is then simulated through the connected river–lake network by accounting for (i) upstream contributions from WWTP

with different treatment levels, (ii) dilution by river discharge, (iii) temperature-dependent pathogen decay during river transport, (iv) attenuation in lakes based on a complete-mixing mass balance including temperature-dependent decay and ultraviolet (UV) radiation inactivation during lake residence time, and (v) flow-weighted mixing at river confluences.

In addition, for river bathing sites, pathogen concentrations are resolved at the specific site location along a river segment by simulating within-segment transport and local WWTP inputs. For bathing sites located in lakes, a lake-wide concentration is first calculated and then adjusted near inflows using a simplified plume-mixing approach.

Data Sources and Variables

The model relies on hydrological and wastewater-related parameters extracted from global hydrographic datasets, primarily RiverATLAS (derived from HydroRIVERS), HydroWASTE, and HydroLAKES[25,27–30]. Table A3 summarizes the key input variables used in the fate model.

Table A3: Model input variables (described below)

Name	Description	Source	Units
Q_{river}	Average river discharge	RiverATLAS	$m^3\ s^{-1}$
L	River reach length	RiverATLAS	km
V_{river}	River reach volume	RiverATLAS	m^3
A_{lake}	Lake surface area	HydroLAKES	km^2
D_{lake}	Average lake depth	HydroLAKES	m
V_{lake}	Lake volume	HydroLAKES	m^3
Q_{out}	Lake outflow discharge	HydroLAKES	m^3/s
Q_{WWTP}	Wastewater effluent discharge	HydroWASTE	m^3/day
LEVEL	WWTP treatment level	HydroWASTE	-
V_{river}	River volume	HydroRIVERS	$10^3\ m^3$
τ_{lake}	Lake residence time	HydroLAKES	days

Advection within Rivers and Lakes

Hydrological Network Representation

The river network is represented as a series of connected river reaches derived from the RiverATLAS database[28]. For each target river segment, all upstream segments are identified recursively using the network connectivity information. This recursive procedure allows the model to trace pathogen contributions from upstream sources, including both upstream river segments and WWTP discharging along the network.

Recursive Aggregation of Upstream Contributions For a given target river segment, the pathogen concentration entering the segment from upstream (C_{up}) is calculated by aggregating the contributions from all immediately upstream segments. This is achieved using a recursive traversal of the river network, which identifies all upstream segments until headwater reaches without upstream connections are reached.

At river confluences, pathogen concentrations from upstream tributaries are combined using a flow-weighted mixing approach:

$$C_{\mathrm{up}} = \frac{\sum_{i=1}^{n} Q_{\mathrm{river},i} \cdot C_{\mathrm{final},i}}{\sum_{i=1}^{n} Q_{\mathrm{river},i}} \tag{9}$$

where $Q_{\mathrm{river},i}$ represents the average discharge of the ith upstream river segment and $C_{\mathrm{final},i}$ is the pathogen concentration at the downstream end of that upstream segment.

The resulting concentration C_{up} represents the pathogen concentration entering the target river segment before additional inputs from local WWTP and within-segment transport processes are considered.

Pathogen decay

The pathogen decay can be described in two parts[65]: temperature- and light-dependent decay. Both decays are integrated in the model by the following Eq. 10:

$$C_{site} = C_{mixed}\ \exp(-k_{\text{UV}}\,\tau_{lake} - k_{\text{dark}}\,t)\,, \tag{10}$$

where

C_{site}	= final pathogen concentration at the bating site [$\#\,\text{L}^{-1}$],
C_{mixed}	= pathogen concentration after WWTP aggregation [$\#\,\text{L}^{-1}$],
k_{UV}	= decay rate constant due to UV radiation [time^{-1}],
τ_{lake}	= lake residence time [day],
k_{dark}	= dark decay rate constant [time^{-1}],
t	= exposure time of dark decay [time].

Pathogen fate in rivers

Temperature-dependent decay

During transport along the river, it is assumed that pathogens are only subject to temperature-dependent decay. The exposure time, which is equivalent to the pathogen's travel time through a river segment of length L (km), is computed as:

$$t = \frac{L}{u}, \quad u = \frac{Q_{\text{river}}}{1000 A_{\text{river}}} \tag{11}$$

where t is the exposure time (day), u is the mean flow velocity (km/day), Q_{river} is river discharge (m^3/day), and A_{river} is the cross-sectional area of the river (m^2)

The decay rate k_{dark} (day^{-1}) is determined following a temperature-dependent first-order model employed in a QMRA study conducted by Schijven et al. [64]:

$$k_{dark} = \frac{\ln(10)}{10^{a_0 + a_1 T}} \tag{12}$$

where a_0 and a_1 are pathogen-specific decay coefficients, whose values are extracted from the same study and shown in Table A4. T is the mean water temperature ($^\circ$ C) derived from

the selected Swiss FOEN monitoring stations inSwitzerland during the peak bathing seasons (June – August)[66], which acts as an approximation of the average water temperature for the fate model.

Table A4: Pathogen-specific model parameters for temperature-dependent decay[64].

**Pathogen**	a_0	a_1
E. coli	1.04	−0.017
Campylobacter jejuni	0.53	−0.017
Norovirus	2.30	−0.036
Adenovirus	2.10	−0.036
Rotavirus	1.60	−0.036
Cryptosporidium parvum	3.10	−0.078

Pathogen fate in lakes

Pathogen attenuation in lakes is modeled using a three-step approach: (i) estimation of the actively mixed surface layer, (ii) calculation of pathogen concentrations using a steady-state mass balance that accounts for inflows, wastewater inputs, and temperature-dependent decay, and (iii) additional inactivation due to ultraviolet (UV) radiation during the lake residence time.

Surface Mixed Layers Volume Following Wells et al.[31], the surface mixed layer in a lake is estimated based on its surface area and its active mixing depth Z_{mixed}. Wells et al. found that the surface mixed layer in a lake is correlated with its fetch (km), which is the distance over which the wind can blow over a lake[31]. The fetch can be empirically estimated using the lake surface area:

$$\text{Fetch} = \sqrt{A_{\text{lake}}} \tag{13}$$

The Z_{mixed} of the lake is computed as:

$$Z_{\text{mixed}} = 4\sqrt{Fetch} \tag{14}$$

The lower value between Z_{mixed} and the mean lake depth (D) is taken as the actual surface mixing depth of the lake:

$$Z_{\text{mixed}} = \min(D_{\text{lake}}, Z_{\text{mixed}}) \tag{15}$$

As a result, the volume of the surface mixed layer was:

$$V_{\text{mixed}} = Z_{\text{mixed}} \cdot A_{\text{lake}} \tag{16}$$

Mass Balance and Decay Assuming complete mixing within V_{mixed} and steady state conditions, the concentration of pathogens in the lake is calculated using a mass balance that accounts for the inflow of pathogens from the river segments leading to the lake, temperature-based pathogen decay, WWTP effluents that discharge directly into the lake, the lake outflow and the accumulation of pathogens.

$$V_{mixed}\frac{dC_{lake,temp\ dacay}}{dt} = \sum C_0 \cdot Q_{\text{WWTP}} + \sum C_{\text{in}} \cdot Q_{\text{in}} - C_{lake} \cdot Q_{\text{out}} - (\mu \cdot V_{\text{mixed}} \cdot C_{lake,temp\ decay}) \tag{17}$$

Here, C_0 again represents the pathogen concentration of a WWTP effluent discharging into the lake, and C_{in} is the calculated concentration of each river segment that flows into the lake. Q_{out} is lake outflow discharge and μ is the temperature-dependent decay rate calculated in Eq. 12. Under steady-state conditions, the accumulation term is zero $(V_{mixed}\frac{dC}{dt} = 0)$. Solution for the concentration of pathogens in the lake C_{lake} is then,

$$C_{\text{lake,temp decay}} = \frac{\sum C_0 \cdot Q_{\text{WWTP}} + \sum C_{\text{in}} \cdot Q_{\text{in}}}{Q_{\text{out}} + \mu \cdot V_{\text{mixed}}} \tag{18}$$

UV radiation-dependent decay As UV radiation also contributes to pathogen decay[65], we use daily UV radiation data 6 Swiss stations reported in TEMIS[32] to model the pathogen decay due to UV-B radiation. We only model the UV-B related inactivation for lakes due to their long residence time. In order to account for the turbidity of the lake water, we collected the Secchi depth based on the concept of Tyler[33] from 12 lakes reported in datalakes[37]. As described by Poole-Atkins relation[67], the attenuation coefficient (Kd) of photosynthetically active radiation (PAR) can be calculated via:

$$K_{d,\mathrm{PAR}} = \frac{1.7}{\overline{z}_{\mathrm{sd}}}, \qquad K_{d,\mathrm{UVB}} = \gamma K_{d,\mathrm{PAR}}, \quad \gamma = 2.0 \tag{19}$$

where $\overline{z}_{\mathrm{sd}}$ = mean Secchi depth across all valid observations [m],
γ = conversion factor from PAR to UVB attenuation [–].

To account for the reduction of UV-B light with depth, the model calculates the effective UV dose with the following equation, following the procedure by[65]:

$$H_{\mathrm{eff}} = f_{\mathrm{surf}} \left(\frac{1 - e^{-Kd(UVB)\, z_m}}{Kd(UVB)\, z_m} \right) H \tag{20}$$

where

H_{eff} = effective (depth-averaged) UV dose [$\mathrm{J\,m^{-2}}$],

H = surface UV dose (e.g., cloud-modified DNA-damage dose) [$\mathrm{J\,m^{-2}}$],

f_{surf} = fraction of pathogens in the sunlit surface layer [–],

$Kd(PAR)$ = diffuse attenuation coefficient for UVB [$\mathrm{m^{-1}}$],

z_m = exposure or mixing depth defining the sunlit layer [m].

The Z_{mixed} of lakes and the depth of rivers are used to estimate the depth-dependent radiation. Based on the cumulated energy doses, the pathogen-specific inactivation rates (Table A5) are used to calculate the first-order decay rate k_{UV} (day^{-1}) due to UV radiation during the residence time with Eq. 21.

$$k_{\mathrm{UV}} = k_{\mathrm{H}} \cdot H_{\mathrm{eff}} \tag{21}$$

The pathogen's concentration in the lake subjected to decay by UV radiation, which is also the final concentration of that lake (C_{lake}), can then be calculated as follows for the duration of the lake's residence time τ_{lake},

$$C_{lake} = C_{lake,temp\ decay} \cdot e^{-k_{\text{UV}} \cdot \tau_{\text{lake}}} \tag{22}$$

Table A5: UV inactivation rate constants (k_{H}) for selected pathogens.

**Pathogen**	k_{H} **(m²/J)**	**Ref.**
E. coli	3.5×10^{-7}	[68]
Campylobacter jejuni	1.93×10^{-6}	[68]
Norovirus	5.6×10^{-7}	[69]
Adenovirus	3.5×10^{-4}	[70]
Rotavirus	3.43×10^{-3}	[71]
Cryptosporidium parvum	1.13×10^{-5}	[72]

Adjustment for inflow plume zones The lake mass balance described above assumes complete mixing within the surface mixed layer and therefore yields a spatially uniform lake concentration. However, river inflows may locally increase pathogen concentrations near the lake entry point before full mixing occurs.

To account for this effect, bathing sites located near river inflows are adjusted using a simplified plume-mixing approach. For each bathing site, the distance to the nearest inflowing river segment is calculated. If the bathing site is located within a predefined mixing distance from the inflow, the site-specific concentration is estimated as a weighted mixture of the inflow concentration and the background lake concentration:

$$C_{\text{site}} = w(d)C_{\text{inflow}} + (1 - w(d))C_{\text{lake}} \tag{23}$$

where C_{inflow} is the pathogen concentration of the inflowing river, C_{lake} is the lake-wide concentration obtained from the mass balance model, and $w(d)$ is a distance-dependent weighting factor that decreases with increasing distance d from the inflow.

Beyond the defined mixing distance, bathing sites are assumed to experience the fully mixed lake concentration.

Calculation of Site-Specific Pathogen Concentrations

Bathing sites are linked to the nearest river segment in the hydrological network. Site-specific pathogen concentrations are derived depending on whether the bathing site is located along a river or within a lake.

For river bathing sites, pathogen concentrations are calculated along the corresponding river segment using a within-segment transport simulation. The concentration entering the segment (C_{up}) is first obtained from upstream tributaries using the recursive aggregation procedure described above. Local inputs from WWTP located along the segment are incorporated at their projected positions assuming instantaneous mixing with the river flow. Pathogen transport downstream is modeled assuming advection with first-order decay. The concentration profile along the river segment is then used to determine the pathogen concentration at the exact location of each bathing site.

For bathing sites located in lakes, concentrations are derived from the lake attenuation model described in Section "Pathogen fate in lakes". The lake-wide concentration obtained from the steady-state mass balance represents the background concentration in the mixed lake layer. For bathing sites located close to river inflows, concentrations are further adjusted using the plume-mixing approach described above, which accounts for locally elevated pathogen concentrations near inflowing rivers before full mixing occurs.

The workflow for calculating site-specific pathogen concentrations is illustrated in Figure A1.

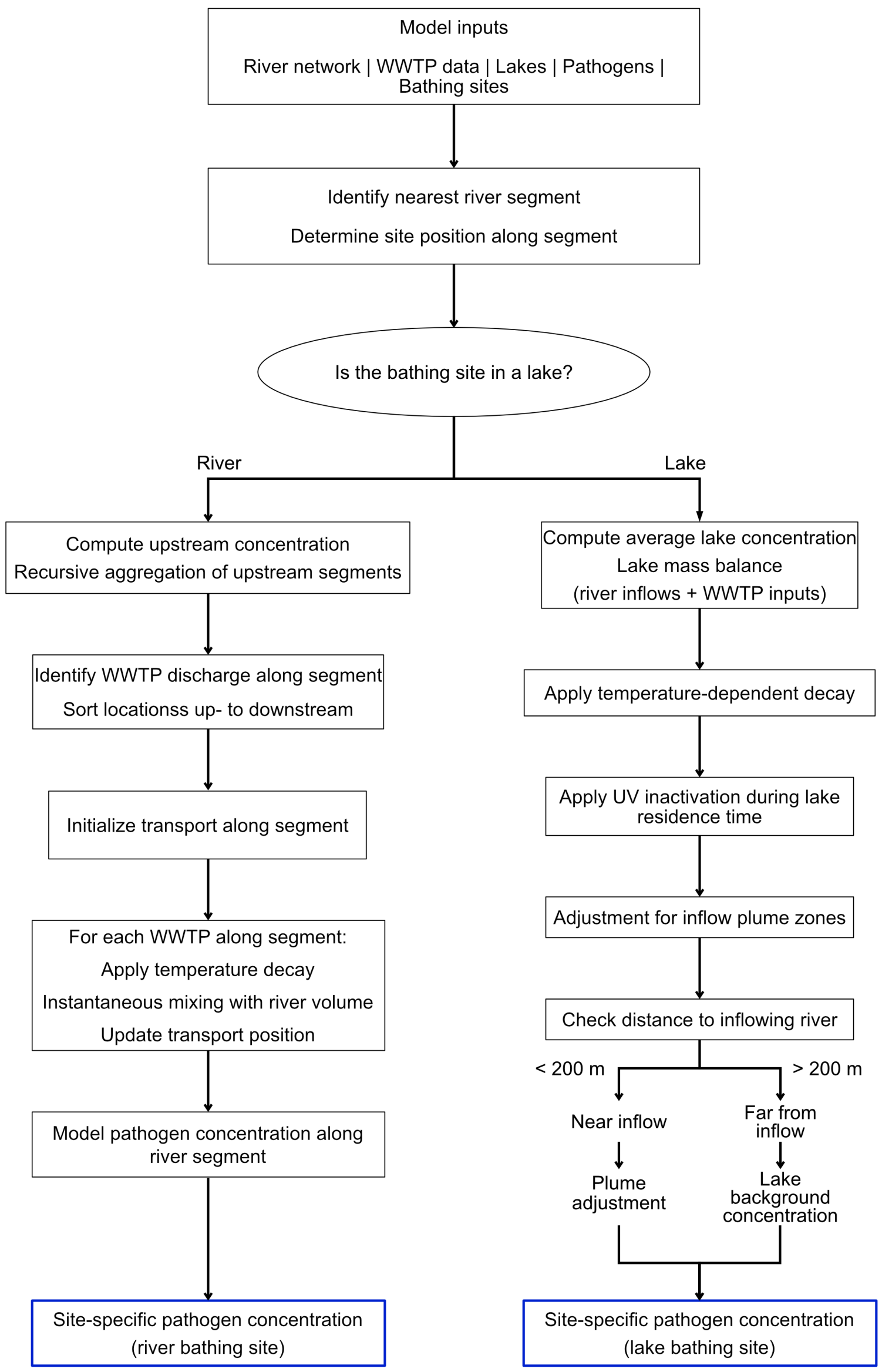

Figure A1: Flowchart of the algorithm for computing the site-specific pathogen concentration on a river segment.

Overview of assessed hydrological network

Table A6 and A7 present a summary of the hydrological network in Switzerland which is based on the RiverATLAS[30].

Table A6: Summary statistics of river segment lengths derived from RiverATLAS.

Metric	n	Mean (km)	Median (km)	p05 (km)	p95 (km)	Min (km)	Max (km)	Sum (km)
River segment length	7899	1.69	1.26	0.10	4.73	4.56×10^{-5}	14.19	13321.19

Table A7: Summary statistics of lake areas derived from RiverATLAS.

Metric	n	Mean (km^2)	Median (km^2)	p05 (km^2)	p95 (km^2)	Min (km^2)	Max (km^2)	Sum (km^2)
Lake area	882	1.57	0.03	0.011	1.40	0.000146	339.95	1388.16

Pathogen concentrations in Switzerland

Tables A8, A9, and A10 present the pathogen concentrations across Swiss rivers and lakes.

Table A8: Pathogen concentration statistics across Swiss rivers and lakes. Summary statistics exclude zero-concentration entries (n = 19,310 total locations).

**Pathogen**	**Min**	**P5**	**Median**	**P95**	**Max**	n **(non-zero)**	n **(zero)**
Adenovirus	1.4×10^{-31}	1.9×10^{-12}	3.2×10^{-2}	9.2×10^{-1}	3.1×10^{1}	7,173	12,137
C. jejuni	4.3×10^{-9}	6.7×10^{-6}	8.1×10^{-3}	5.6×10^{-1}	2.2×10^{1}	7,173	12,137
Crypto. parvum	1.6×10^{-10}	3.0×10^{-8}	1.1×10^{-3}	6.2×10^{-2}	1.8×10^{0}	7,173	12,137
E. coli	1.2×10^{-3}	1.7×10^{0}	6.4×10^{2}	4.3×10^{4}	1.5×10^{6}	7,173	12,137
Norovirus	1.0×10^{-8}	1.6×10^{-5}	2.1×10^{-3}	5.0×10^{-2}	1.5×10^{0}	7,173	12,137
Rotavirus	6.9×10^{-277}	9.7×10^{-87}	4.9×10^{-5}	1.5×10^{-3}	5.0×10^{-2}	7,173	12,137

Figures A2 - A6 show the spatial variability of the modeled concentrations for the assessed pathogens in this study.

Table A9: Pathogen concentration statistics for river bathing sites (Switzerland). Statistics exclude zero-concentration entries ($n = 108$ total rows).

**Pathogen**	**Min**	**P5**	**Median**	**P95**	**Max**	n **(non-zero)**	n **(zero)**
Adenovirus	2.4×10^{-13}	2.4×10^{-13}	1.6×10^{-1}	1.4×10^{0}	1.7×10^{0}	93	15
C. jejuni	8.0×10^{-6}	4.7×10^{-5}	9.1×10^{-2}	8.8×10^{-1}	1.1×10^{0}	93	15
Crypto. parvum	1.3×10^{-6}	1.5×10^{-5}	1.0×10^{-2}	8.0×10^{-2}	1.0×10^{-1}	93	15
E. coli	1.8×10^{0}	1.2×10^{1}	6.7×10^{3}	6.3×10^{4}	7.9×10^{4}	93	15
Norovirus	1.6×10^{-5}	1.1×10^{-4}	8.1×10^{-3}	6.5×10^{-2}	8.1×10^{-2}	93	15
Rotavirus	4.2×10^{-103}	4.2×10^{-103}	2.4×10^{-4}	2.2×10^{-3}	2.7×10^{-3}	93	15

Table A10: Pathogen concentration statistics for lake bathing sites (Switzerland). Statistics exclude zero-concentration entries ($n = 395$ total rows).

**Pathogen**	**Min**	**P5**	**Median**	**P95**	**Max**	n **(non-zero)**	n **(zero)**
Adenovirus	2.4×10^{-13}	2.4×10^{-13}	8.6×10^{-5}	5.5×10^{-1}	7.7×10^{0}	245	150
C. jejuni	1.4×10^{-6}	8.0×10^{-6}	1.4×10^{-3}	3.6×10^{-1}	5.4×10^{0}	245	150
Crypto. parvum	3.0×10^{-8}	2.7×10^{-7}	7.4×10^{-5}	3.1×10^{-2}	4.5×10^{-1}	245	150
E. coli	3.1×10^{-1}	1.8×10^{0}	3.1×10^{2}	2.5×10^{4}	3.7×10^{5}	245	150
Norovirus	1.6×10^{-5}	1.6×10^{-5}	1.6×10^{-3}	2.6×10^{-2}	3.6×10^{-1}	245	150
Rotavirus	4.2×10^{-103}	4.2×10^{-103}	7.0×10^{-25}	8.7×10^{-4}	1.2×10^{-2}	245	150

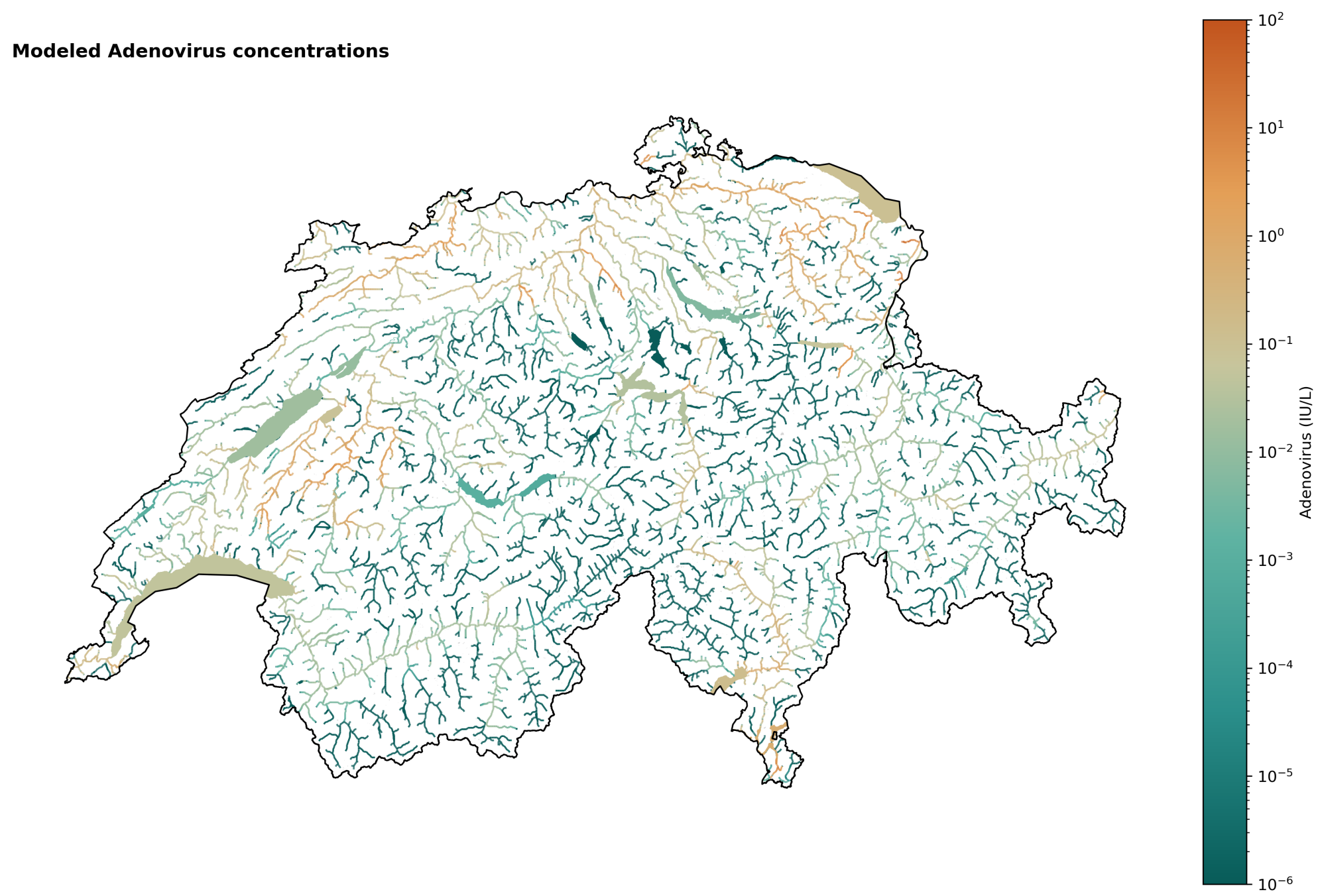

Figure A2: Modeled Adenovirus concentration expressed in Infectious Units per L.

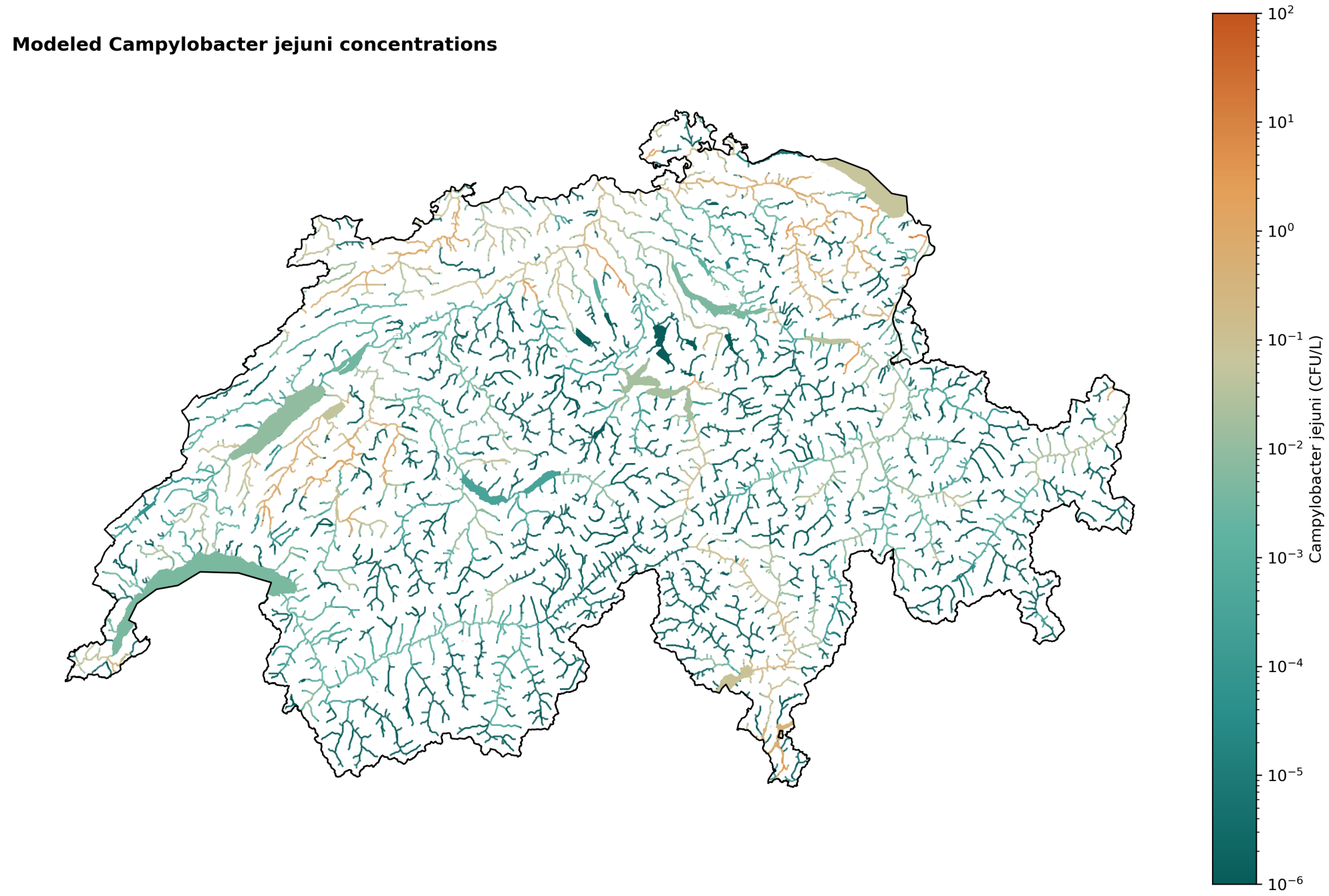

Figure A3: Modeled Campylobacter jejuni concentration expressed in CFU per L.

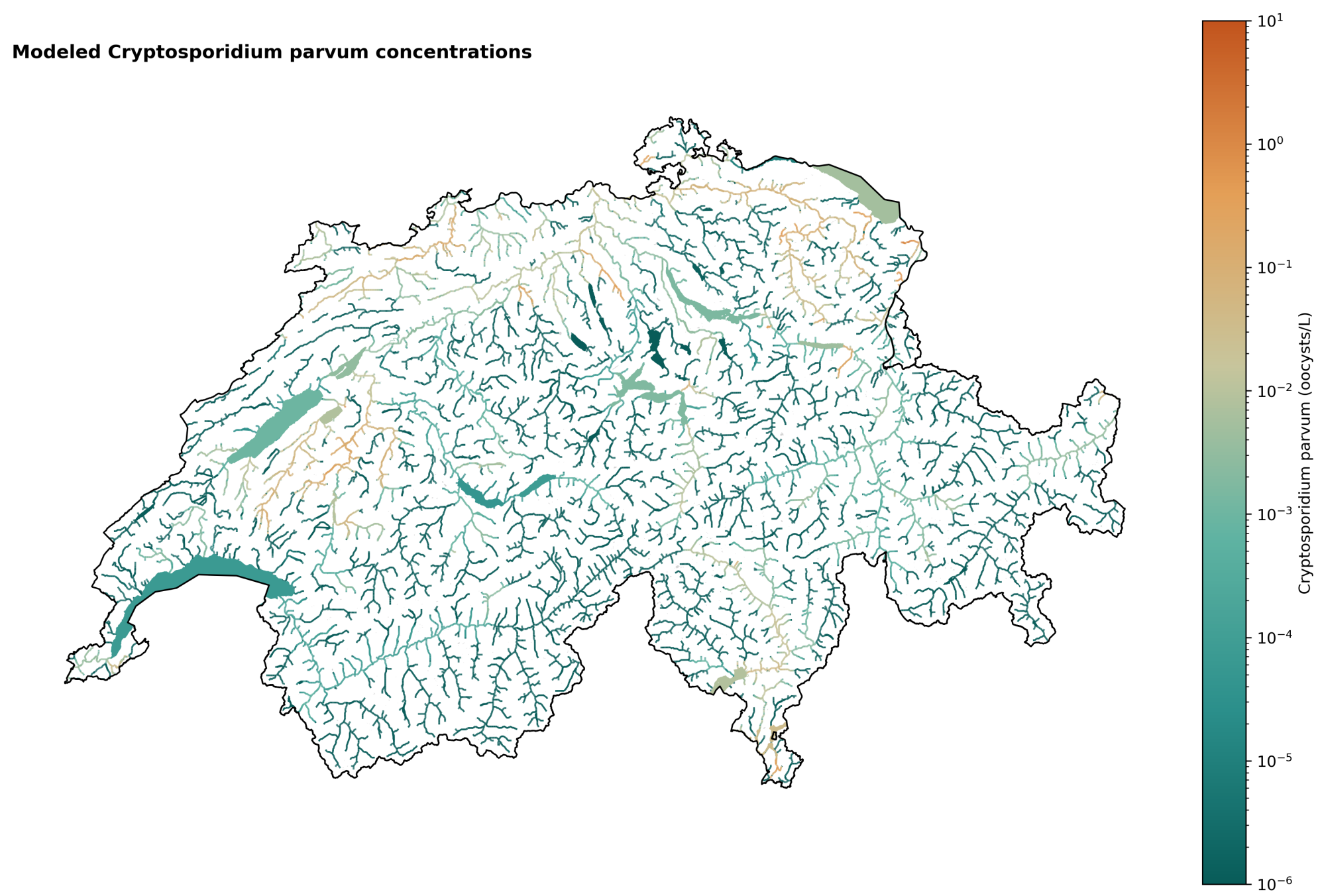

Figure A4: Modeled Cryptosporidium parvum concentration expressed in oocysts per L.

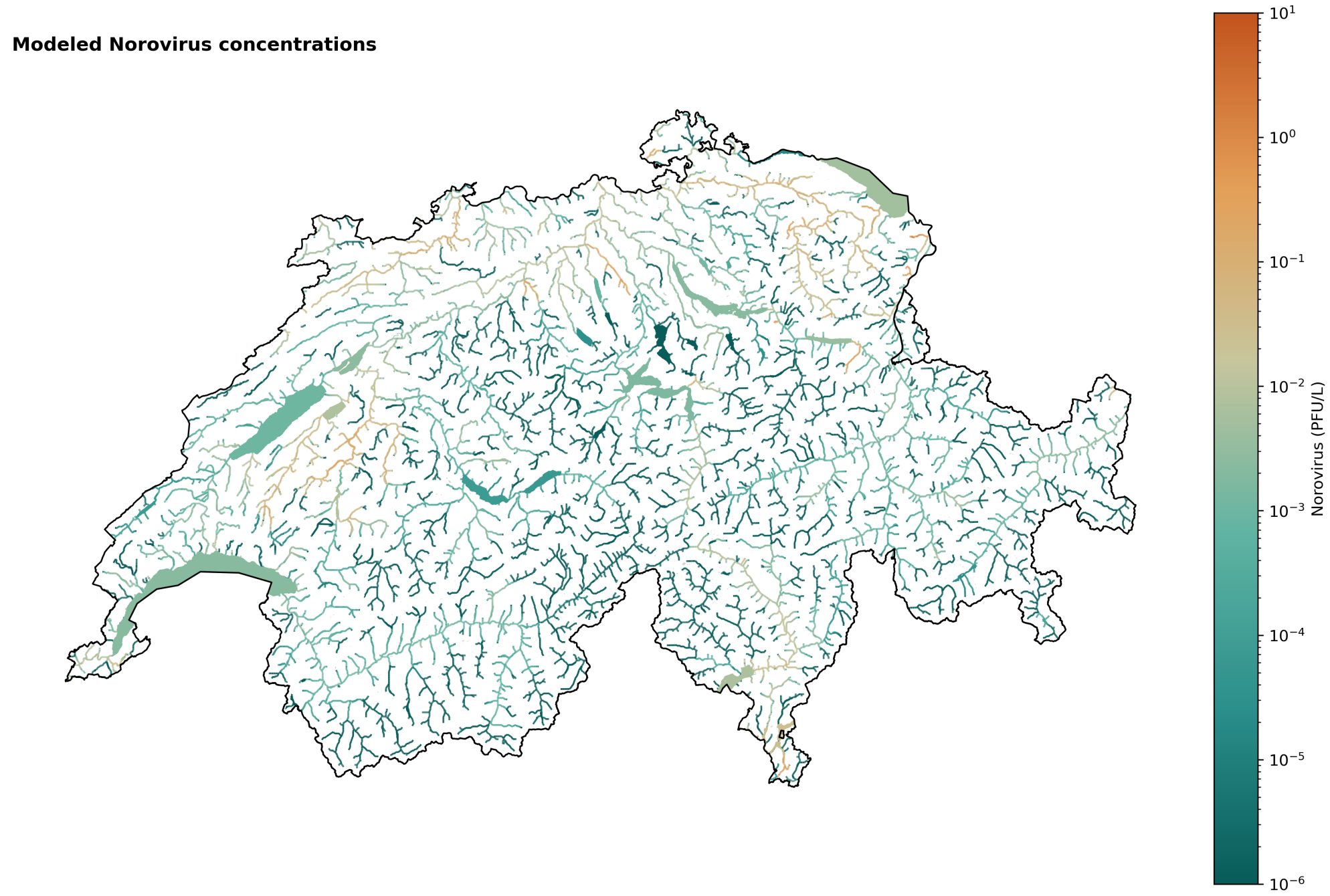

Figure A5: Modeled Norovirus concentration expressed in PFU per L.

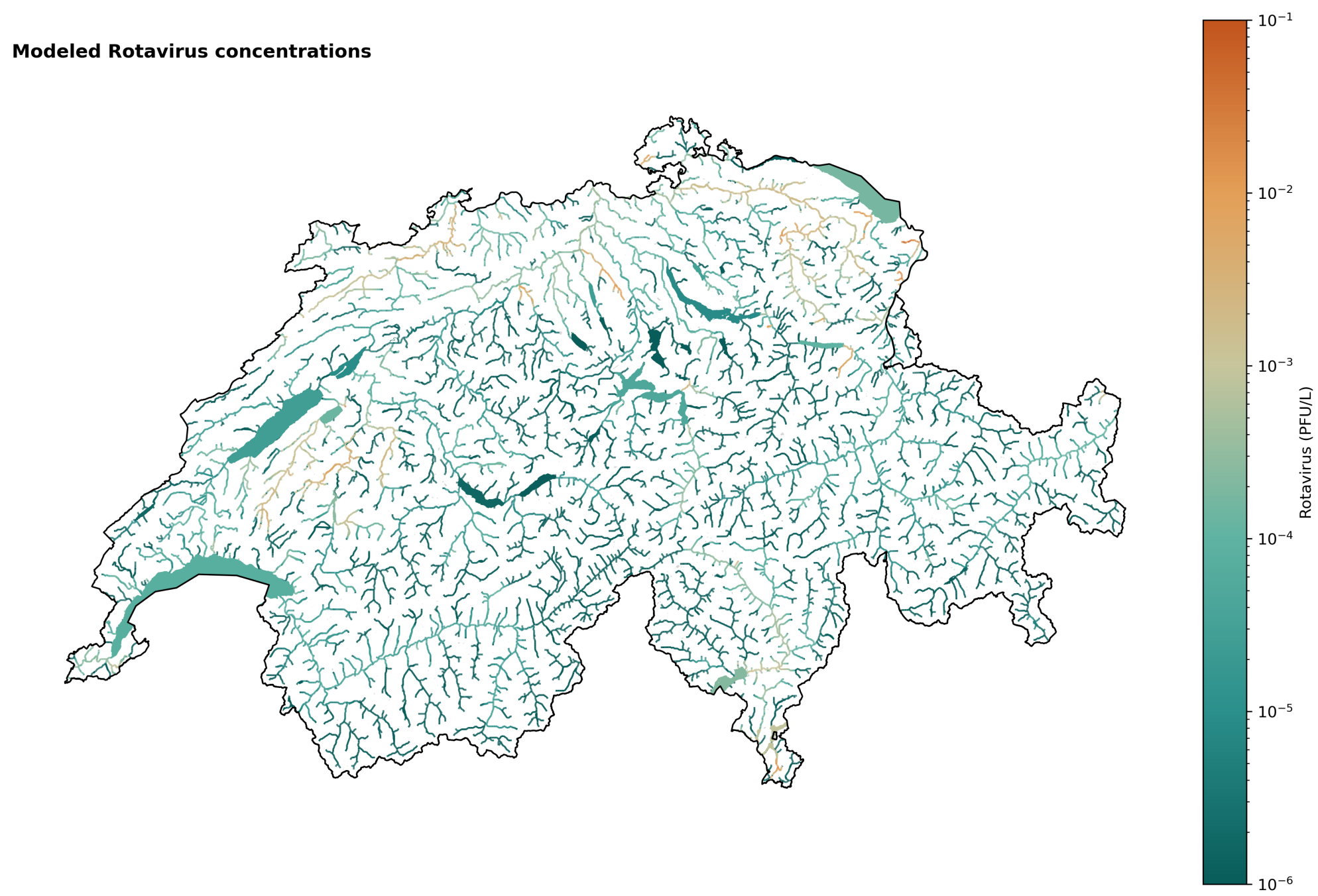

Figure A6: Modeled Rotavirus concentration expressed in PFU per L.

Model Validation

Model predictions of E. coli concentrations are evaluated against observational data from official bathing water monitoring programs in Switzerland, from cantons of Zurich, Thurgau and Ticino[73–75]. For each available bathing site, an empirical concentration distribution is constructed using all available measurements from the earliest recorded year through 2025, restricted to the bathing season (June–August). Observed zero concentrations are replaced by a small positive value (0.1) for visualization purposes.

For river systems, validation is performed at spatially resolved bathing-site locations and observed site-specific concentration distributions are compared against model predictions of the corresponding bathing sites (Figure A7). Model predictions generally fall within the observed ranges, although some overestimation can be observed from the model for Rhein and Thur. The model also captures the differences among sites located along the same river - sites downstream from WWTP show higher modeled concentrations due to cumulative pathogen loads and/or high river velocities than the sites located upstream from WWTP - while the measured concentrations do not show this variability. Occasionally the model overestimates site concentrations (e.g. at 'Thur #2').

For lakes, similar validation is performed using modeled lake buffer zones, which accounts for the concentration gradients due to localized influences of nearby WWTP discharges. Bathing sites representative of the lake buffer zones are chosen, and observed and modeled concentrations are extracted accordingly (Figure A8). Half of the model predictions broadly align with observed medians at bathing locations. Similar to rivers, the model predictions are influenced by local emission sources and thus show considerable spatial variability within the same lake, which is not evident in the observational data.

Figures A9 and A10 compare both the mean and median of the model predictions against aggregated observed distributions at the water-body scale. It should be noted that the cantonal monitoring data are available only as concentration measurements, without meteorological or hydrological information during the measurements. Therefore, short-term

influences (e.g., rainfall, flooding, etc.) at the time of sampling cannot be accounted for in the model–data comparison. What's more, plant specific measurements with influent and effluent wastewater concentrations, as well as measurements from various locations along a single river segment would be very helpful to a more accurate model validation.

The global minimum and maximum of modeled concentration are also compared with literature values in Table A11. The modeled concentrations generally fall below the range of literature-reported values, and the discrepancies are largely due to the possibly conservative assumption on the influent pathogen concentrations in the model.

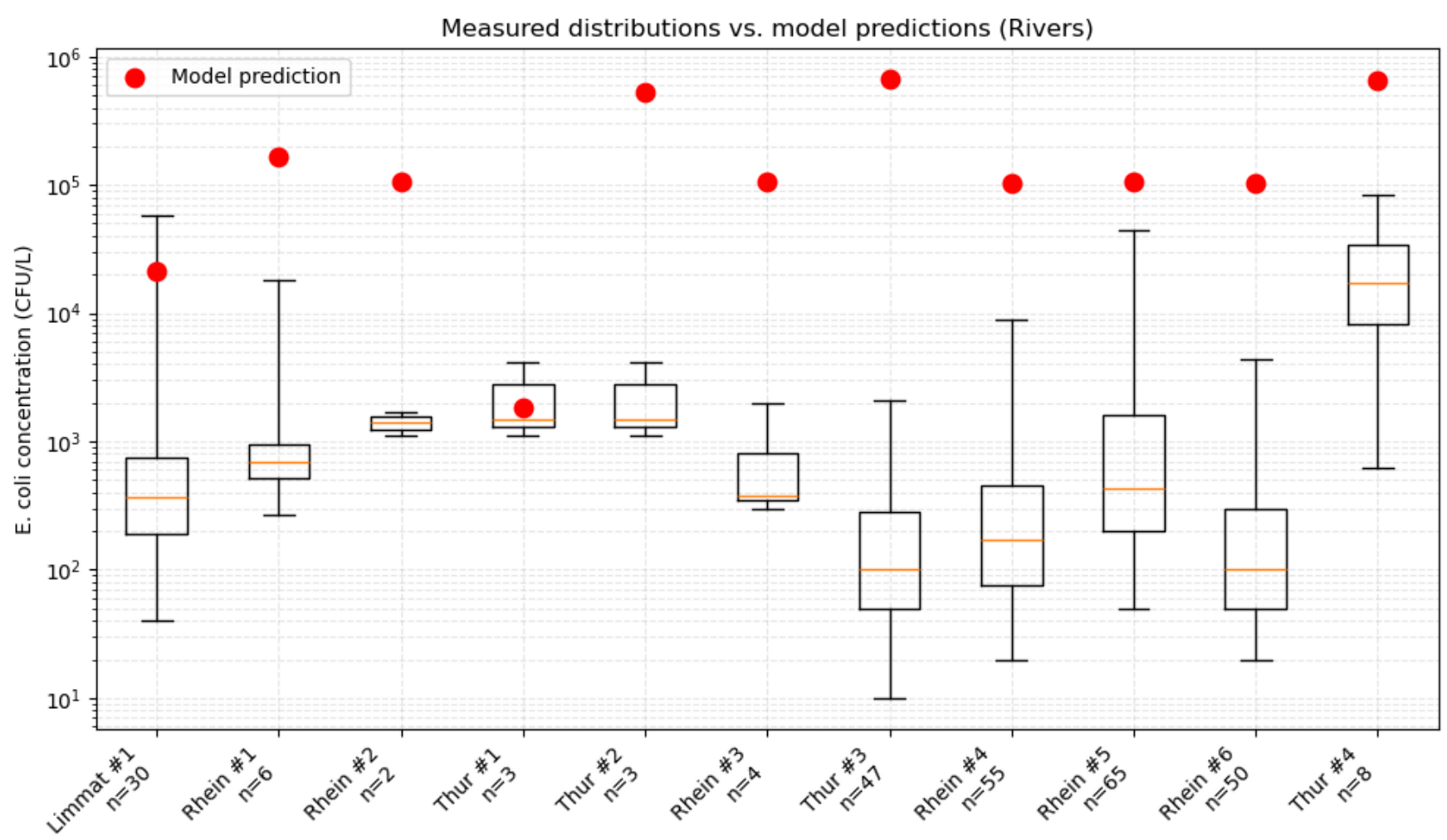

Figure A7: Spatially resolved comparison between measured *E. coli* concentration distributions and model predictions for selected river bathing sites. Each boxplot corresponds to one monitoring location: top and bottom of the box indicate the interquartile range (25th–75th percentile), with the horizontal line representing the median; lower and upper whiskers correspond to the minimum and maximum observed concentrations; sample sizes (n) are indicated by the number of individual measurement points contributing to each distribution; red points indicate site-specific model predictions.

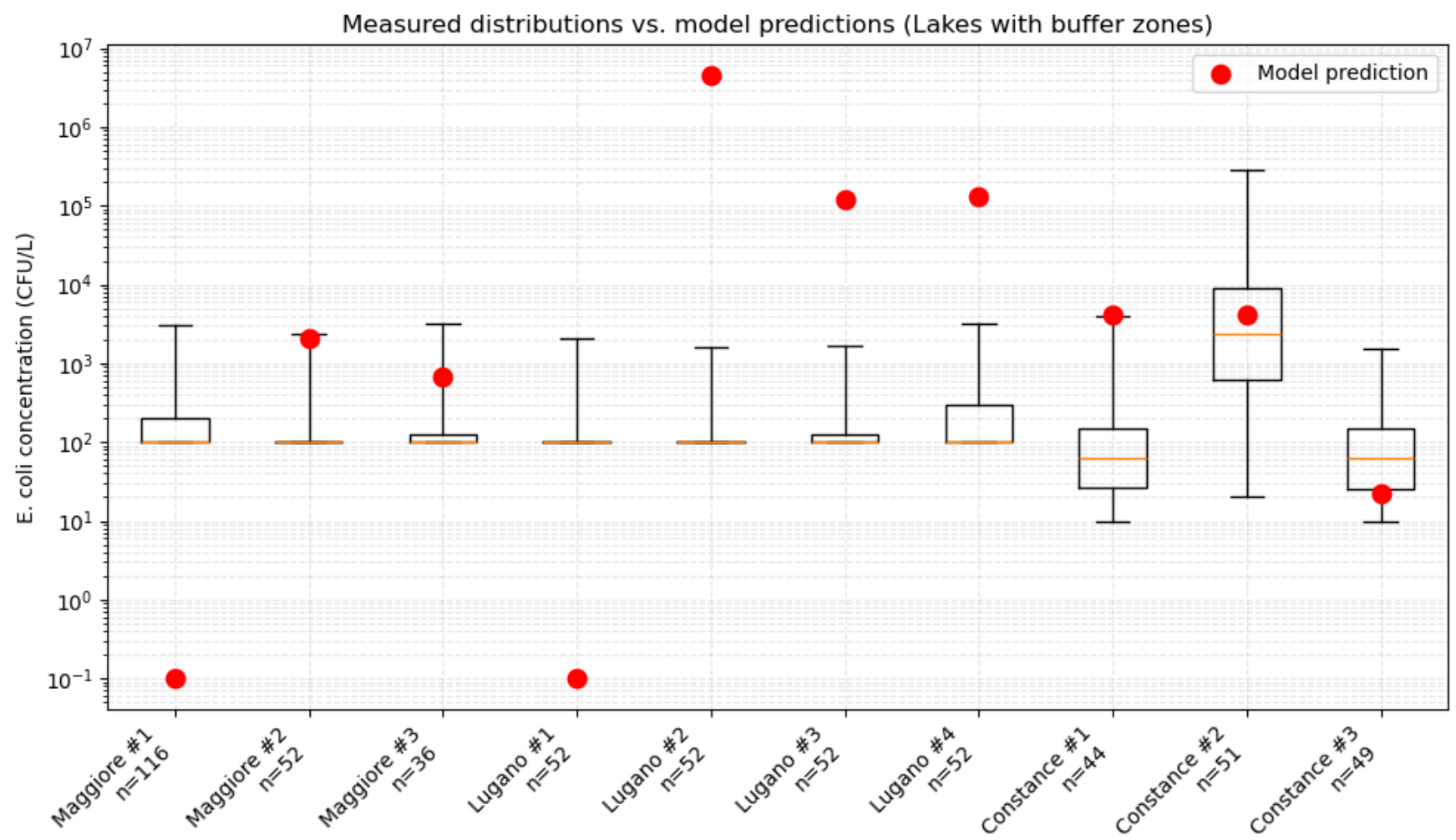

Figure A8: Spatially resolved comparison between measured *E. coli* concentration distributions and model predictions for selected lake bathing sites. Each boxplot corresponds to one monitoring location: top and bottom of the box indicate the interquartile range (25th–75th percentile), with the horizontal line representing the median; lower and upper whiskers correspond to the minimum and maximum observed concentrations; sample sizes (n) are indicated by the number of individual measurement points contributing to each distribution; red points indicate site-specific model predictions.

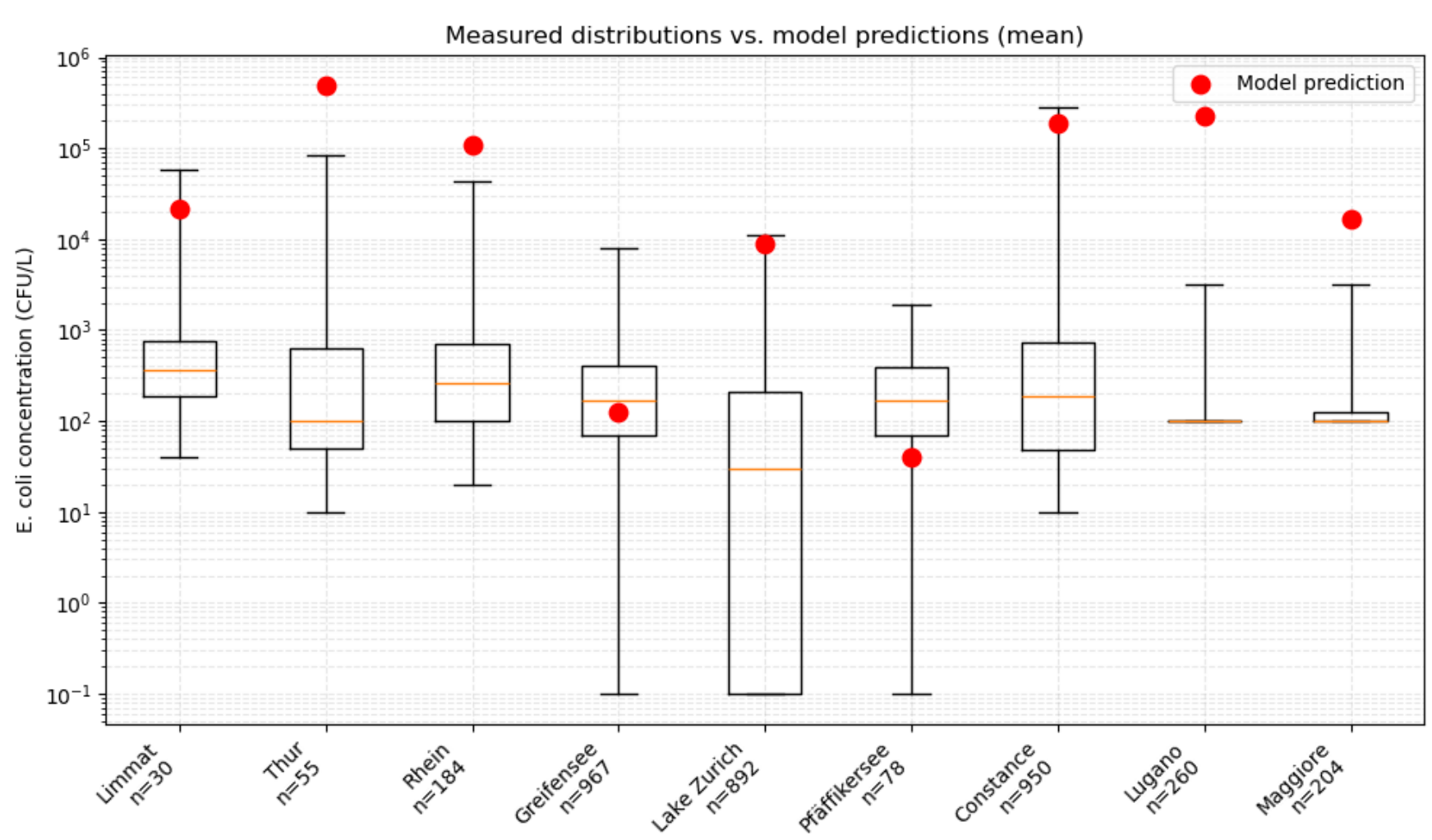

Figure A9: Comparison between measured *E. coli* concentration distributions and model predictions aggregated by water body using the arithmetic mean of modeled concentrations. Boxplots summarise observed bathing-water measurements: top and bottom of the box indicate the interquartile range (25th–75th percentile), with the horizontal line representing the median; lower and upper whiskers correspond to the minimum and maximum observed concentrations; sample sizes (n) are indicated by the number of individual measurement points contributing to each distribution; red points indicate model predictions.

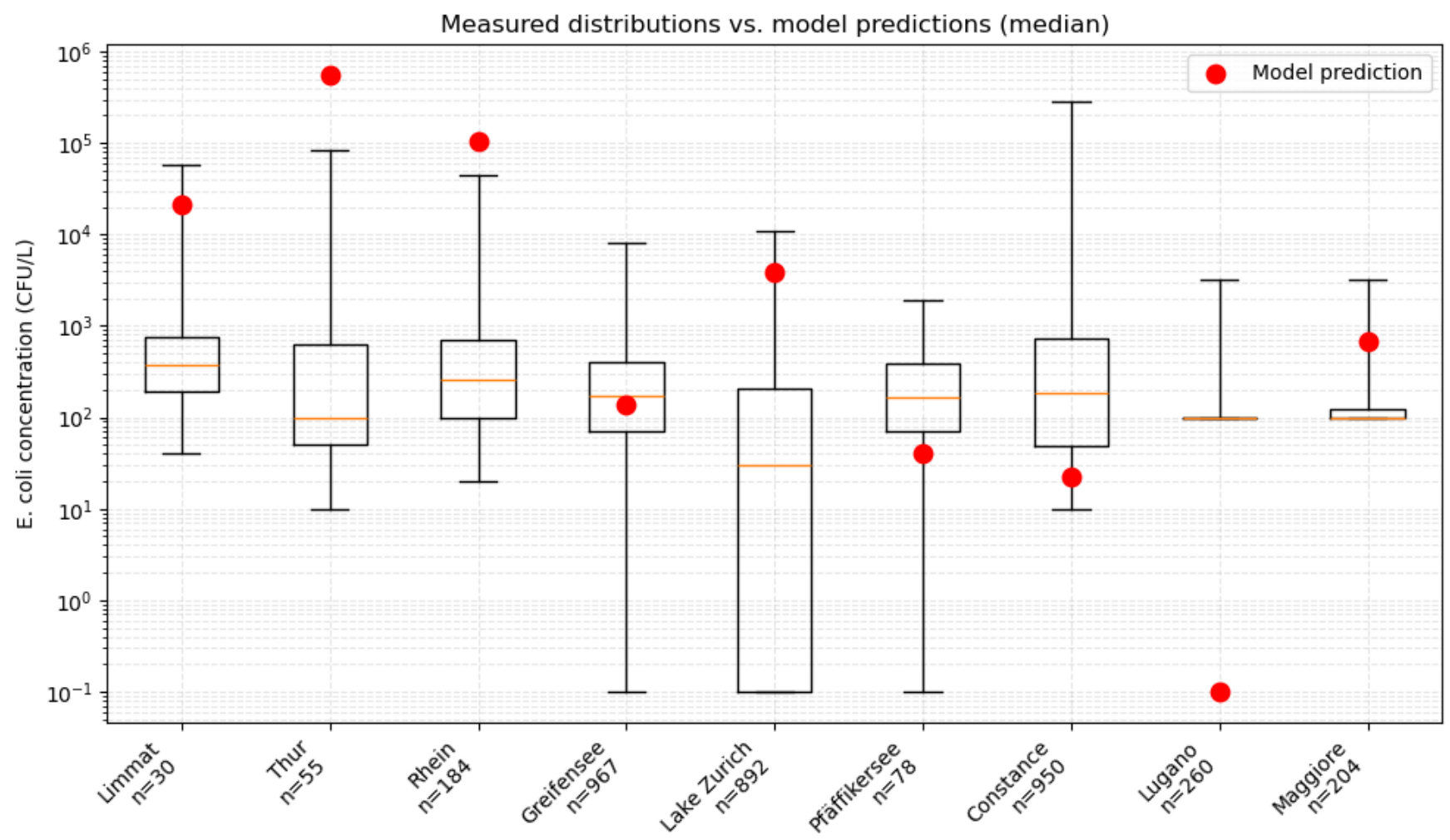

Figure A10: Comparison between measured *E. coli* concentration distributions and model predictions aggregated by water body using the median of modeled concentrations. Boxplots summarise observed bathing-water measurements: top and bottom of the box indicate the interquartile range (25th–75th percentile), with the horizontal line representing the median; lower and upper whiskers correspond to the minimum and maximum observed concentrations; sample sizes (n) are indicated by the number of individual measurement points contributing to each distribution; red points indicate model predictions.

Table A11: Modeled concentration ranges at bathing sites in Switzerland compared with representative mean concentrations reported in the literature of rivers and lakes across Europe, North and South America.

**Pathogen**	**Min modeled**	**Max modeled**	**Literature values**	**Unit**	**Ref.**
Campylobacter jejuni	1.36×10^{-6}	5.43	30	CFU L^{-1}	[76]
Norovirus	1.60×10^{-5}	3.63×10^{-1}	3.00×10^{3}	GC L^{-1}	[77]
Adenovirus	2.38×10^{-13}	7.71	10^{3}–10^{4}	IU L^{-1}	[77,78]
Rotavirus	4.20×10^{-103}	1.23×10^{-2}	15	PFU L^{-1}	[79]
Cryptosporidium parvum	2.97×10^{-8}	4.49×10^{-1}	52	oocysts L^{-1}	[80]

Exposure

The population bands are only assigned to one recreational sites in order to avoid any double-counting. For the Swiss case, we observe that some regions report only a limited number of recreational sites (e.g., Berne). Once more data become available, the model can be used to quantify the exposure of these new sites. The mean of the frequency (Figure A11) and uptake (Figure A12) are used to determine doses.

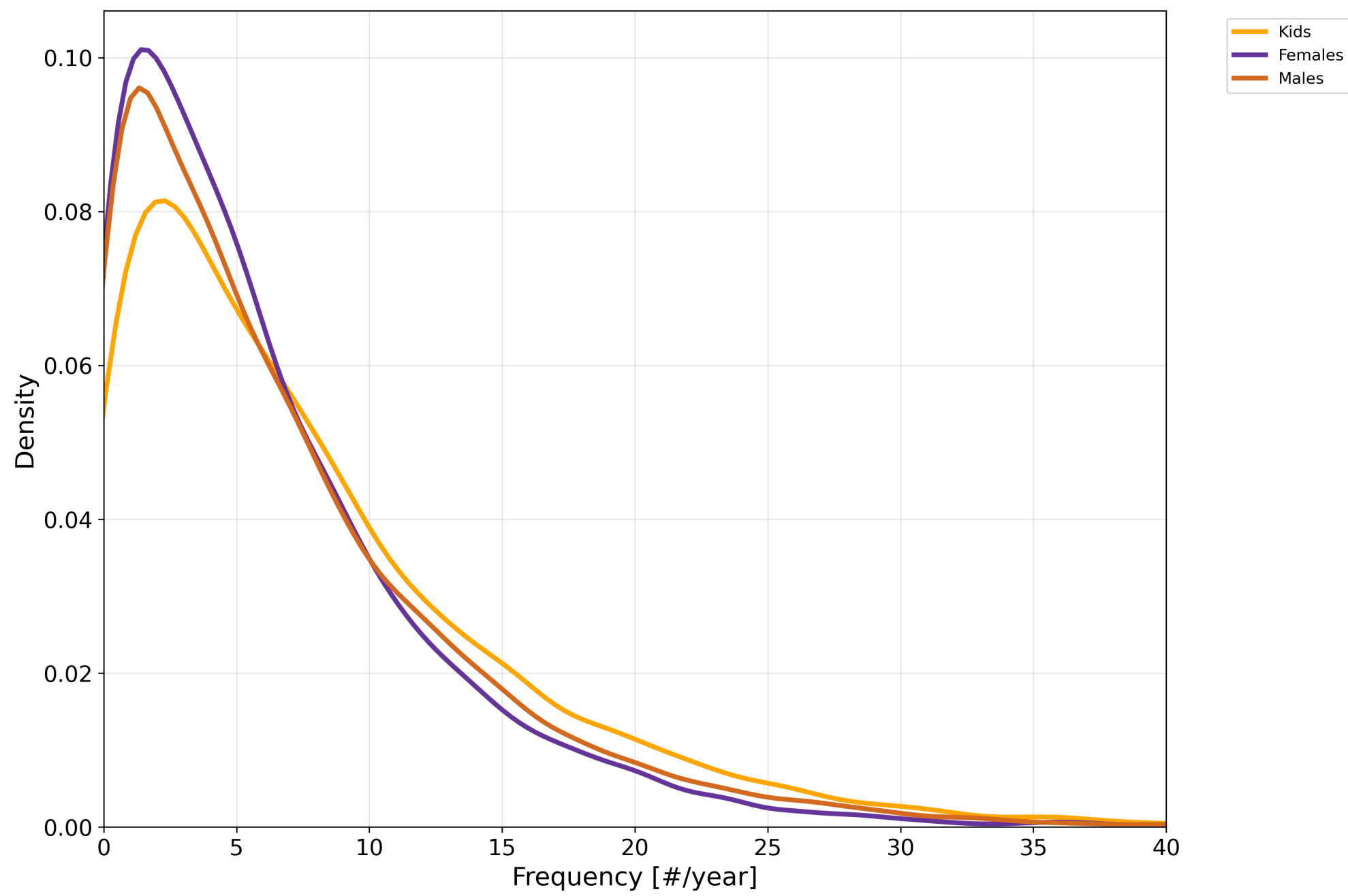

Figure A11: Frequency distribution for the aggregated population groups divided in females, males and kids. N = 10000

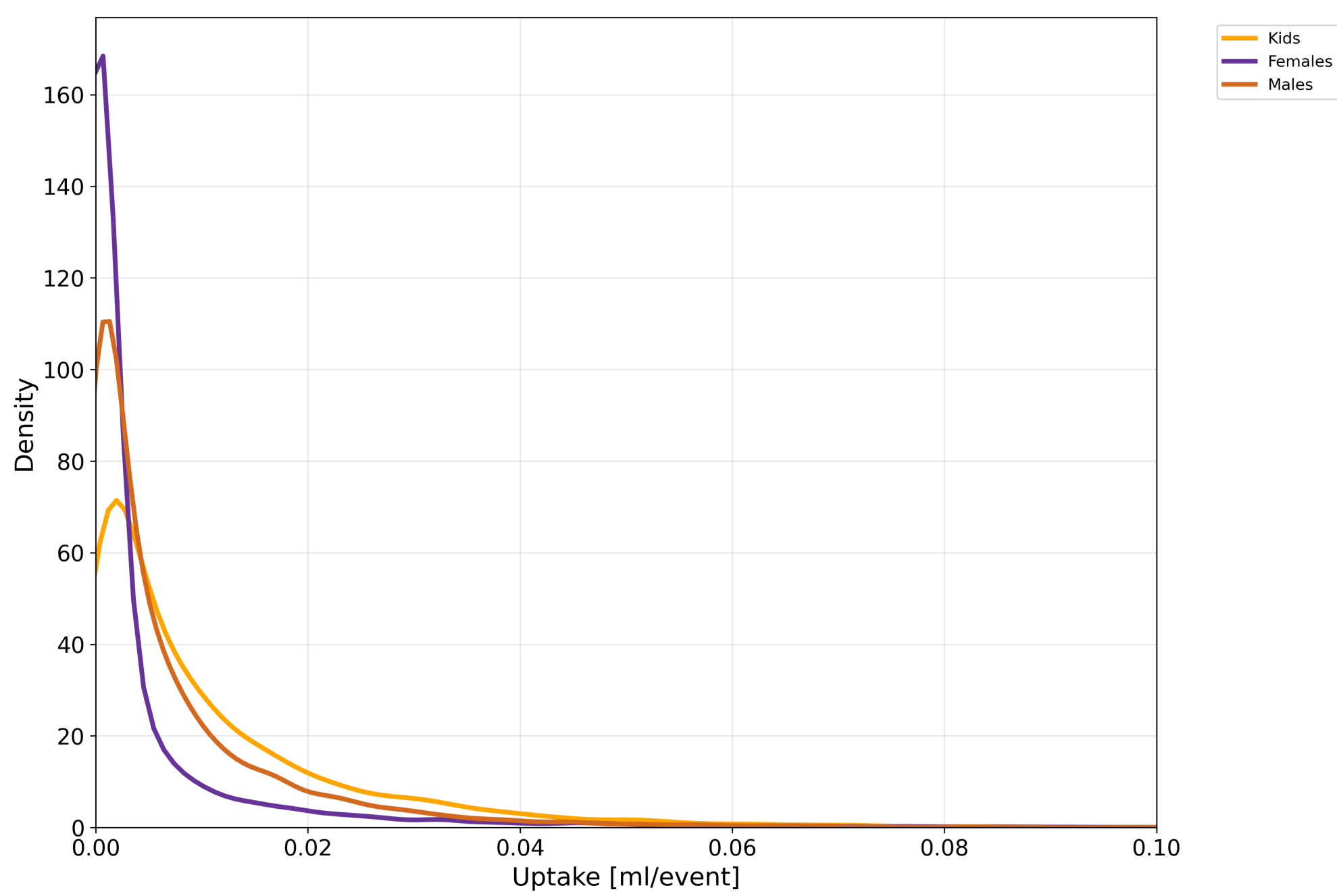

Figure A12: Water uptake distribution for the aggregated population groups divided in females, males and kids. N = 10000

Dose response

The chosen dose response curves are reported in Table A12. The estimated infections also include infections which can be often asymptomatic and need to be taken into account. Besides that, several dose response curves (i.e., E. coli) are modeled with the response type being diarrhea. Hence, the different endpoints of dose response curves need to be normalized. The model applies the infection-to-illness ratios which are reported for various pathogens (Table A12). Additionally, it should be emphasized that the dose response curves are not adapted for sex, age, or pre-existing diseases lowering the immunity.

Table A12: Dose–response models and parameters of pathogens assessed in this study.

**Pathogen**	**Reference**	**Strain/data source**	**Model**	**Unit**	**Response type**	**Illness per infection**	**Parameter**	**Value**
E. coli	QMRAwiki; Tacket et al.[40]	EPEC O127:H6 strain E2348/69	Beta–Poisson	CFU	Diarrhea	1	α	1.600×10^{-1}
							N_{50}	9.980×10^{7}
Campylobacter jejuni	Sales-Ortells and Medema[12]	Human volunteer and raw-milk outbreak data; strain not specified	Hypergeometric	CFU	Infection	0.33	α	2.400×10^{-2}
							β	1.100×10^{-2}
Norovirus	Sales-Ortells and Medema[12]	Norwalk virus inocula 8fIIa and 8fIIb	Hypergeometric	pdU	Infection	0.67	α	4.000×10^{-2}
							β	5.500×10^{-2}
Adenovirus	QMRAwiki; Teunis et al.[41]	AdV4 and AdV7 oral-ingestion challenge data	Beta–Poisson	$TCID_{50}$	Illness	1	α	5.110
							N_{50}	7.400×10^{-1}
Rotavirus	QMRAwiki; Ward et al.[42]	CJN strain (unpassaged)	Beta–Poisson	FFU	Infection	0.57	α	2.530×10^{-1}
							N_{50}	6.170
Cryptosporidium parvum	QMRAwiki; Okhuysen et al.[43]	Moredun isolate; type 2, cervine origin	Beta–Poisson	Oocysts	Diarrhea	1	α	2.700×10^{-1}
							N_{50}	1.680×10^{1}

Quantification of human health impacts

Human health impacts are expressed as disability-adjusted life years (DALYs). Pathogen- and age-specific DALY factors are derived by combining DALY rates from the Global Burden of Disease database[44] with the incidence rates used in the dose-response model overview. GBD DALY rates are reported per 100,000 population for both sexes and for the age groups "under 5" and "all ages". Switzerland is assigned to the GBD super-region "High-income".

For pathogen p and age group a, the DALY factor per illness case is calculated as

$$d_{p,a} = \frac{r^{\mathrm{DALY}}_{p,a,s(c)}}{I_p}, \tag{24}$$

where $r^{\mathrm{DALY}}_{p,a,s(c)}$ is the GBD DALY rate per 100,000 population for the super-region $s(c)$ of country c, and I_p is the pathogen-specific incidence rate per 100,000 population from the dose-response model overview.

Annual DALYs are calculated by multiplying the median annual illness cases by the corresponding DALY factor:

$$\mathrm{DALY}_{i,p,a} = N_{i,p,a} \cdot d_{p,a}, \tag{25}$$

where $N_{i,p,a}$ denotes the median annual number of illness cases at site i for pathogen p and age group a.

Site-level DALYs are obtained by summing across age groups and pathogens:

$$\mathrm{DALY}^{\mathrm{total}}_i = \sum_p \sum_a \mathrm{DALY}_{i,p,a}. \tag{26}$$

National DALYs are calculated by summing total DALYs across all sites:

$$\mathrm{DALY}_{\mathrm{national}} = \sum_i \mathrm{DALY}^{\mathrm{total}}_i. \tag{27}$$

Uncertainty and global sensitivity analyses

Parameter distributions

Table A13: Sensitivity parameters used in the Monte Carlo uncertainty analysis and Sobol global sensitivity analysis.

Parameter / scope	Distribution	Baseline	Lower	Upper	Rationale / note
**Fate and transport**					
Temperature-related decay scale	lognormal	1	0.2	5	Ranges are defined based on Sosah et al. [81] on temperature related decay rates.
UV decay scale	normal	1	0.2	1.2	Ranges defined based on the results by Loeb et al. [69] for Norovirus
Influent concentration scale	lognormal	1	0.5	2	Ranges defined, Lognormal distribution based on Schijven et al. [10]
Treatment remaining-fraction scale	triangular	1	0.8	1.2	Scales the post-treatment remaining fraction (1 - efficiency/100), not the removal percentage. Values >1 increase emitted concentration; values <1 reduce it.
Lake mixed-layer depth scale	lognormal	1	0.5	2	Range taken from Schijven et al. [10], with a wider range as range is reported for rivers and to account for uncertainty in the depth of the lake mixed layer.
Discharge / dilution scale	normal	1	0.01	5	Ranges defined, Normal distribution based on Schijven et al. [10]
**Exposure**					
Water ingestion / uptake scale	triangular	1	0.01	6.0	Reported range of water ingestion for the entire population by Schets et al. [11]
Exposure frequency scale	uniform	1	0.01	4	Reported frequency for freshwater recreational activities by Schets et al. [11]
Population density scale	triangular	1	0.8	1.2	Proxy for local WorldPop gridded population/spatial allocation uncertainty, not national population total uncertainty.

Continued on next page

Table A13: Sensitivity parameters used in the Monte Carlo uncertainty analysis and Sobol global sensitivity analysis (continued).

Parameter	Distribution	Baseline	Lower	Upper	Note
Affected population fraction scale	triangular	0.5	0.0	1.0	Since no behavioral data exist for Switzerland, we use a maximum range to account for this uncertainty
**Infection / dose response**					
Escherichia coli; dose-response α	lognormal	0.155	0.119	0.321	Direct confidence intervals of E. coli from QMRAwiki are used.
Escherichia coli; dose-response N_{50}	lognormal	9.98×10^7	3.25×10^7	2.63×10^8	Direct confidence intervals of E. coli from QMRAwiki are used.
Escherichia coli; dose-response illness per infection	triangular	1	0	1	Due to the lack of data, we use the broadest range possible.
Campylobacter jejuni; dose-response α	triangular	0.024	0.012	0.048	Due to the lack of additional ranges of the defined parameters, we use a factor of 2 for the selected parameters.
Campylobacter jejuni; dose-response β	triangular	0.011	0.0055	0.022	Due to the lack of additional ranges of the defined parameters, we use a factor of 2 for the selected parameters.
Campylobacter jejuni; dose-response illness per infection	triangular	0.33	0	1	Due to the lack of data, we use the broadest range possible.
Norovirus; dose-response α	triangular	0.04	0.02	0.08	Due to the lack of additional ranges of the defined parameters, we use a factor of 2 for the selected parameters.
Norovirus; dose-response β	triangular	0.055	0.0275	0.11	Due to the lack of additional ranges of the defined parameters, we use a factor of 2 for the selected parameters.

Continued on next page

Table A13: Sensitivity parameters used in the Monte Carlo uncertainty analysis and Sobol global sensitivity analysis (continued).

Parameter	Distribution	Baseline	Lower	Upper	Note
Norovirus; dose-response illness per infection	triangular	0.67	0	1	Due to the lack of data, we use the broadest range possible.
Adenovirus; dose-response α	triangular	5.11	2.555	10.22	Due to the lack of additional ranges of the defined parameters, we use a factor of 2 for the selected parameters.
Adenovirus; dose-response N_{50}	triangular	0.74	0.37	1.48	Due to the lack of additional ranges of the defined parameters, we use a factor of 2 for the selected parameters.
Adenovirus; dose-response pathogenicity r	triangular	0.42	0	1	–
Adenovirus; dose-response illness per infection	triangular	1	0	1	Due to the lack of data, we use the broadest range possible.
Rotavirus; dose-response α	triangular	0.248	0.124	0.496	Due to the lack of additional ranges of the defined parameters, we use a factor of 2 for the selected parameters.
Rotavirus; dose-response N_{50}	triangular	6.17	3.085	12.34	Due to the lack of additional ranges of the defined parameters, we use a factor of 2 for the selected parameters.
Rotavirus; dose-response illness per infection	triangular	0.57	0	1	Due to the lack of data, we use the broadest range possible.
Cryptosporidium parvum; dose-response α	triangular	0.27	0.135	0.54	Due to the lack of additional ranges of the defined parameters, we use a factor of 2 for the selected parameters.
Cryptosporidium parvum; dose-response N_{50}	triangular	16.8	8.4	33.6	Due to the lack of additional ranges of the defined parameters, we use a factor of 2 for the selected parameters.
Cryptosporidium parvum; dose-response illness per infection	triangular	1	0	1	Due to the lack of data, we use the broadest range possible.

Continued on next page

Table A13: Sensitivity parameters used in the Monte Carlo uncertainty analysis and Sobol global sensitivity analysis (continued).

Parameter	Distribution	Baseline	Lower	Upper	Note
**DALY / incidence**					
DALY per case multiplier	lognormal	1	0.5	2	Based on the database by Naghavi et al.[44], we select the range based on their defined upper and lower boundaries for E. coli related DALYs.
Escherichia coli; incidence rate scale	uniform	1	0.2	5	Due to the high uncertainties of reported incidences, we use a broad range to account for unreported cases.
Campylobacter jejuni; incidence rate scale	uniform	1	0.2	5	Due to the high uncertainties of reported incidences, we use a broad range to account for unreported cases.
Norovirus; incidence rate scale	uniform	1	0.2	5	Due to the high uncertainties of reported incidences, we use a broad range to account for unreported cases.
Adenovirus; incidence rate scale	uniform	1	0.2	5	Due to the high uncertainties of reported incidences, we use a broad range to account for unreported cases.
Rotavirus; incidence rate scale	uniform	1	0.2	5	Due to the high uncertainties of reported incidences, we use a broad range to account for unreported cases.
Cryptosporidium parvum; incidence rate scale	uniform	1	0.2	5	Due to the high uncertainties of reported incidences, we use a broad range to account for unreported cases.

Uncertainty analysis

Table A14: Summary statistics of the Monte Carlo uncertainty analysis for total annual DALYs.

Statistic	Total annual DALYs
Mean	2’519.28
Median	853.75
Standard deviation	7’231.34
5th percentile	45.59
25th percentile	290.42
75th percentile	2’297.81
95th percentile	9’300.39
Minimum	0.208
Maximum	220’284.55

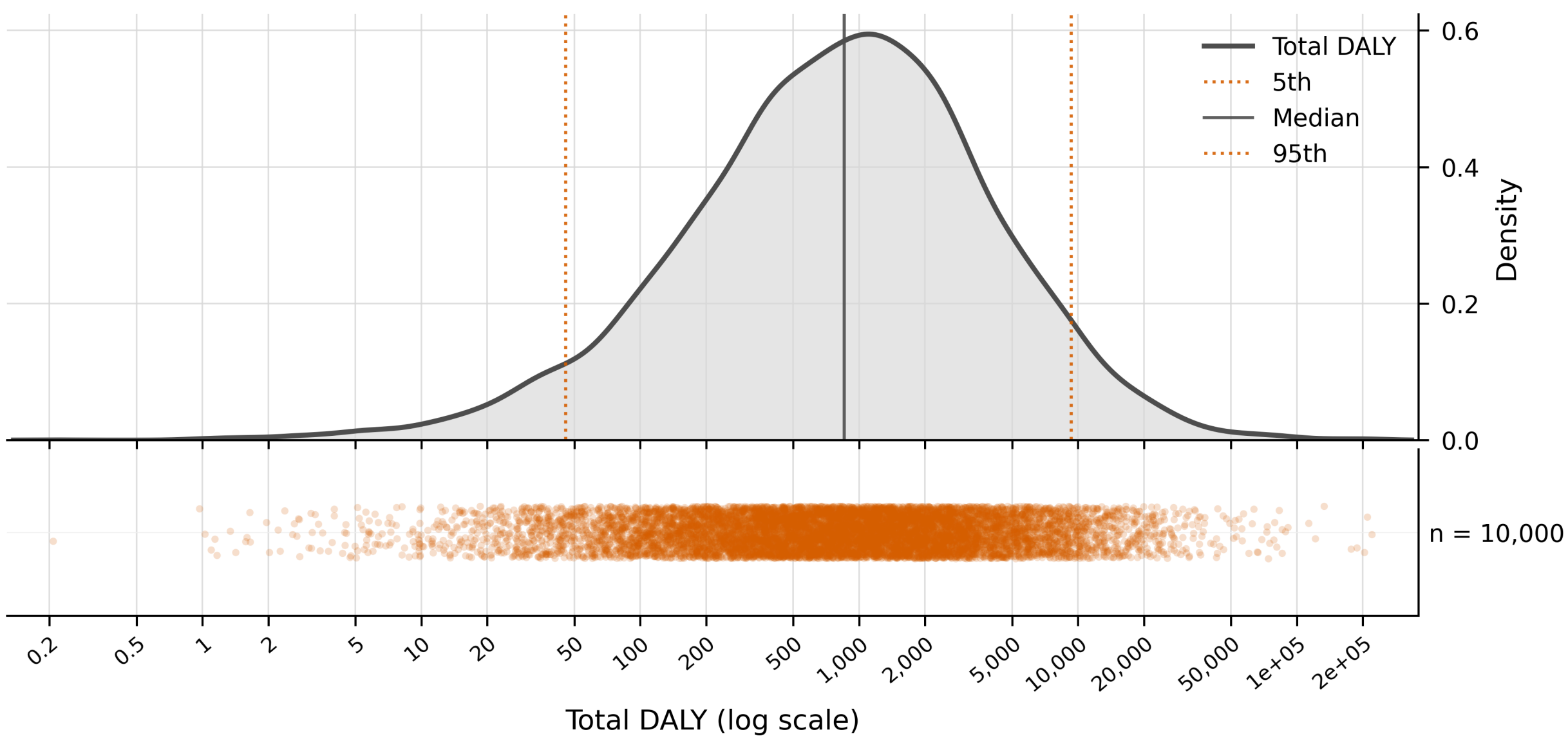

Figure A13: Monte Carlo uncertainty distribution of total annual DALYs. The upper panel shows the kernel density estimate of total DALYs across 10,000 Monte Carlo simulations, with vertical lines indicating the 5th percentile, median, and 95th percentile. The lower panel shows the individual Monte Carlo realizations as jittered points. The x-axis is shown on a logarithmic scale to improve readability of the right-skewed distribution.

Global sensitivity analysis

Table A15: Sobol sensitivity indices for all uncertain parameters. Parameters are ranked by the total-order Sobol index (S_T).

Rank	Parameter	Category	S_1	S_T
1	*E. coli* incidence	Effect	0.162	0.704
2	*E. coli* DR (N50)	Effect	0.0257	0.340
3	Uptake	Exposure	0.0391	0.217
4	*E. coli* DR (α)	Effect	0.0184	0.191
5	Frequency	Exposure	0.117	0.162
6	Affected pop.	Exposure	0.00891	0.0949
7	*E. coli* DR (illness/inf.)	Effect	0.00275	0.0880
8	DALY/case	Effect	0.0180	0.0563
9	Influent conc.	Fate	0.0188	0.0411
10	*Cryptosporidium* DR (α)	Effect	-0.00286	0.0204
11	Pop. size	Exposure	0.00273	0.00739
12	WWTP stage eff.	Fate	0.00371	0.00564
13	T-decay	Fate	0.00196	0.00392
14	*Cryptosporidium* incidence	Effect	0.00320	0.00315
15	Discharge rate	Fate	-0.00208	0.00258
16	*Campylobacter* incidence	Effect	-4.74×10^{-4}	0.00195
17	*Cryptosporidium* DR (N50)	Effect	-2.17×10^{-4}	0.00109
18	*Campylobacter* DR (illness/inf.)	Effect	1.13×10^{-4}	4.85×10^{-4}
19	*Cryptosporidium* DR (illness/inf.)	Effect	-3.12×10^{-4}	3.94×10^{-4}
20	Rotavirus incidence	Effect	1.21×10^{-4}	6.41×10^{-5}
21	Adenovirus incidence	Effect	6.49×10^{-5}	3.15×10^{-5}
22	*Campylobacter* DR (α)	Effect	-3.53×10^{-5}	2.73×10^{-5}
23	Rotavirus DR (α)	Effect	-1.08×10^{-4}	2.65×10^{-5}
24	*Campylobacter* DR (β)	Effect	-3.93×10^{-5}	2.62×10^{-5}
25	Norovirus incidence	Effect	2.51×10^{-5}	2.07×10^{-5}
26	Norovirus DR (illness/inf.)	Effect	-1.86×10^{-5}	2.05×10^{-5}
27	Norovirus DR (α)	Effect	1.17×10^{-4}	1.94×10^{-5}
28	Rotavirus DR (N50)	Effect	1.39×10^{-5}	1.91×10^{-5}
29	Rotavirus DR (illness/inf.)	Effect	1.37×10^{-4}	1.85×10^{-5}
30	Adenovirus DR (illness/inf.)	Effect	-3.80×10^{-5}	1.72×10^{-5}
31	Adenovirus DR (pathogenicity r)	Effect	-6.82×10^{-6}	1.60×10^{-5}
32	Mixing zone	Fate	-9.75×10^{-5}	1.51×10^{-5}
33	UV-decay	Fate	-7.73×10^{-5}	1.29×10^{-5}
34	Adenovirus DR (α)	Effect	-1.11×10^{-4}	1.22×10^{-5}
35	Norovirus DR (β)	Effect	-1.81×10^{-5}	1.09×10^{-5}

Rank	Parameter	Category	S_1	S_T
36	Adenovirus DR (N50)	Effect	2.62×10^{-6}	1.03×10^{-5}

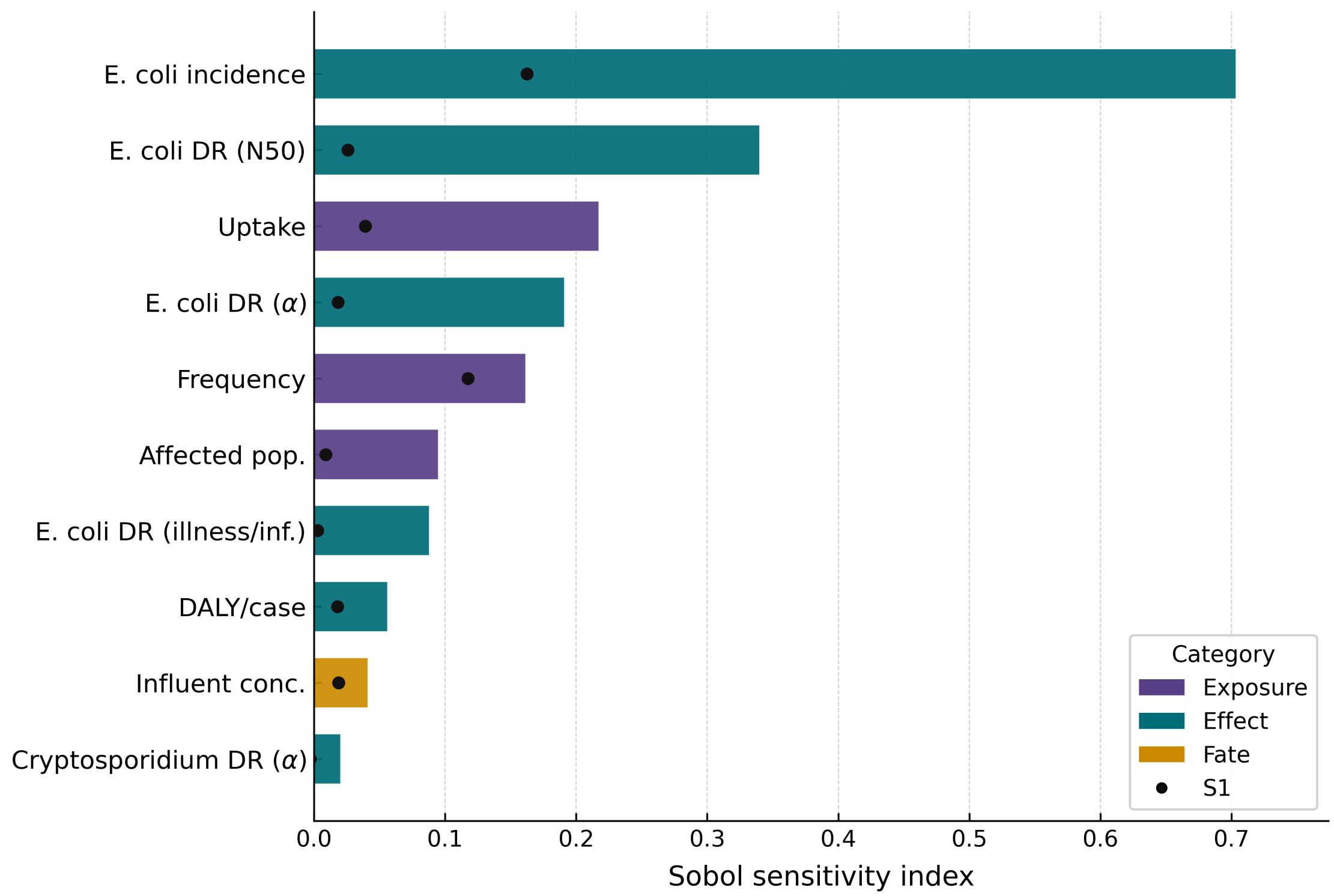

Figure A14: Sobol sensitivity indices for the most influential uncertain parameters in the total annual DALY estimate. Bars show total-order indices (S_T), which include both direct effects and interactions, while black points show first-order indices (S_1). Colours indicate the model component to which each parameter belongs.

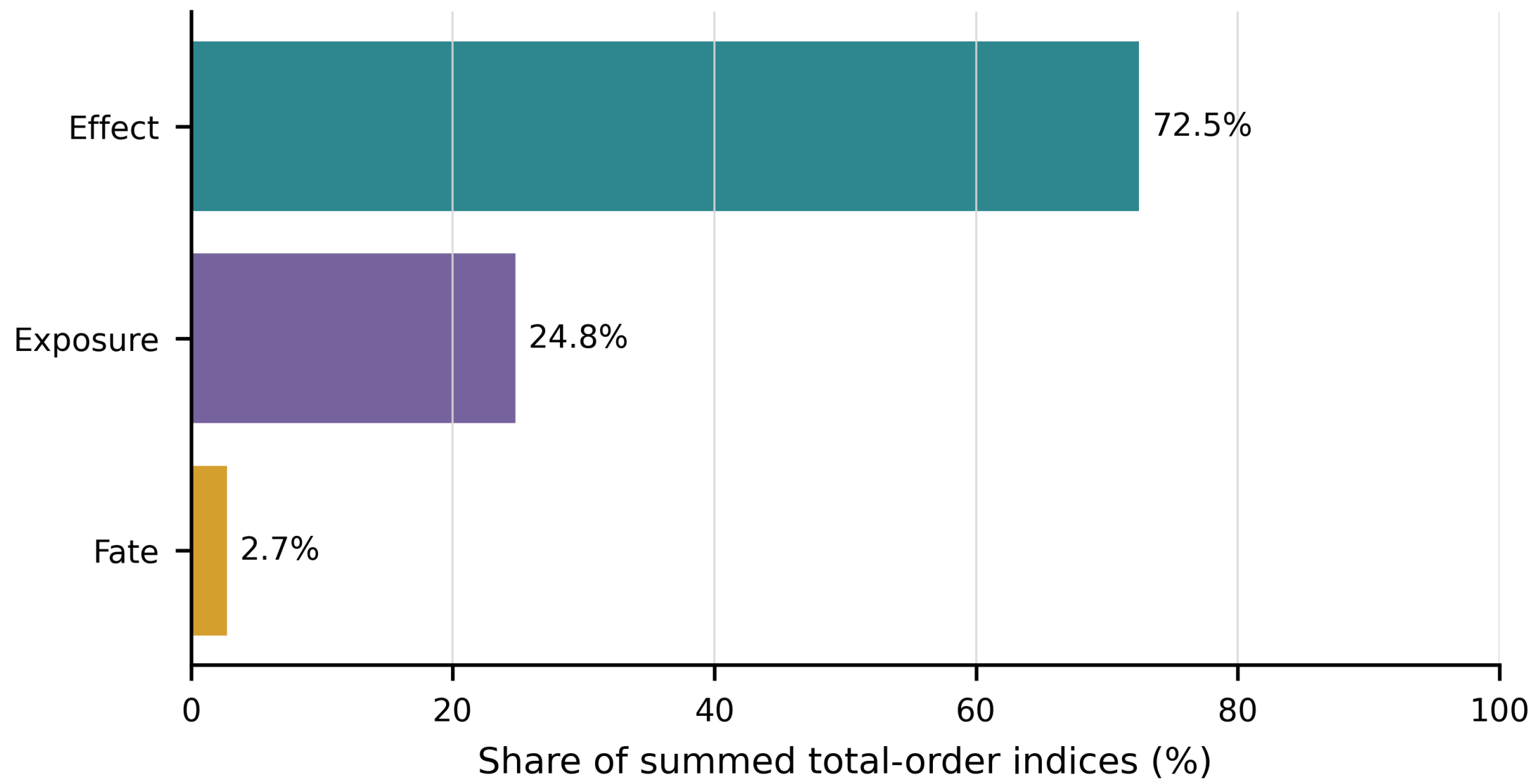

Figure A15: Relative contribution of fate, exposure, and effect parameters to the summed total-order Sobol indices for total annual DALYs.

Treatment stage scenarios

The treatment stage scenarios illustrate how national-scale upgrades in wastewater treatment affect both modeled *E. coli* concentrations at bathing sites and the resulting pathogen-related health burden. Figure A16 shows a clear stepwise decrease in *E. coli* concentrations with increasing treatment level. Concentrations are highest in the no-treatment scenario and remain elevated under primary treatment, although the median is reduced. Secondary treatment leads to a substantial further decrease, while advanced treatment reduces concentrations by several orders of magnitude. The wide spread of points, particularly for no treatment and primary treatment, indicates that some bathing sites remain strongly affected even when the central tendency decreases.

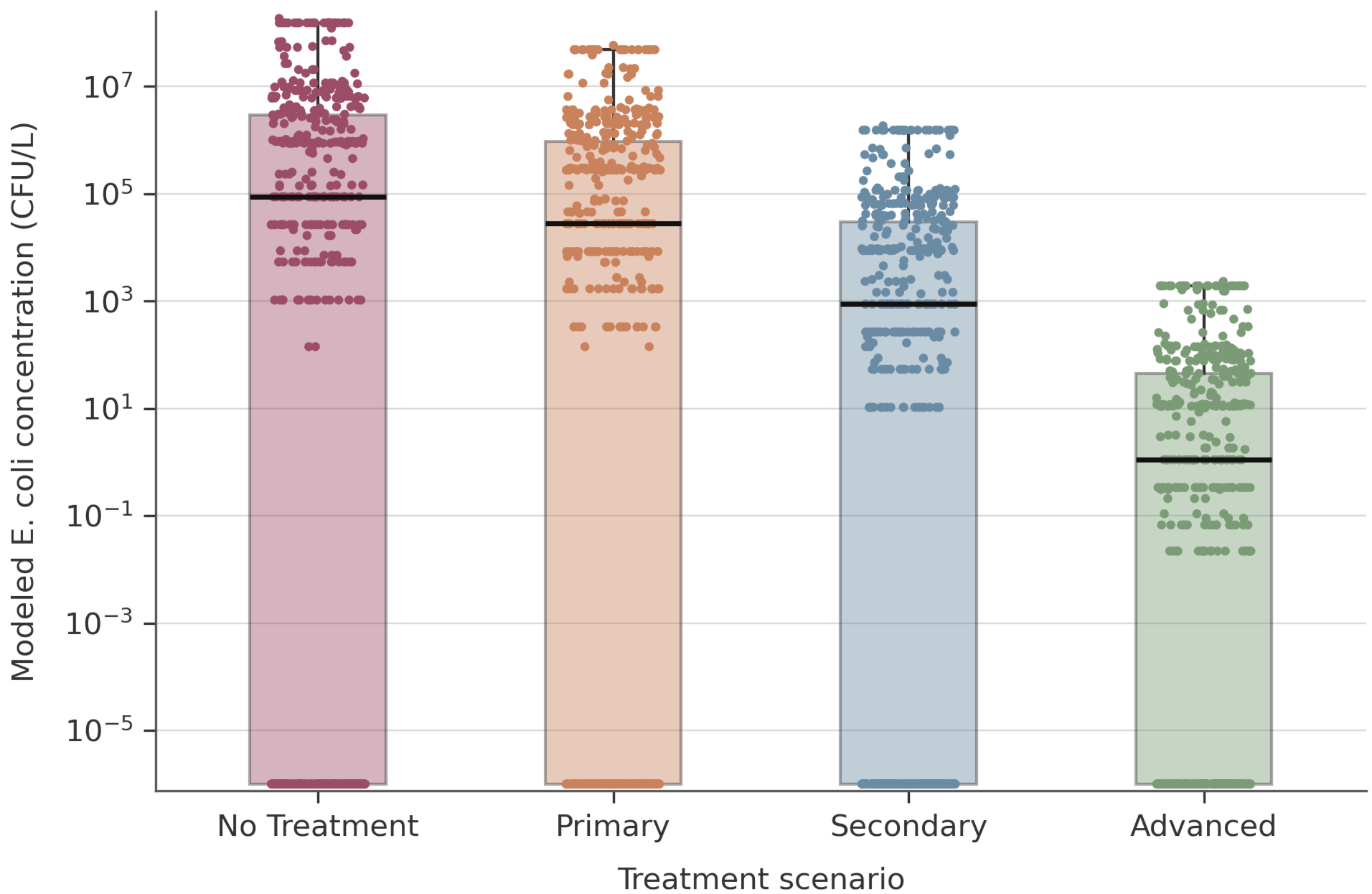

Figure A16: Modeled *E. coli* concentrations at bathing sites across wastewater treatment scenarios. Points represent individual site-level concentrations. Boxes show the interquartile range (25th–75th percentile), the thick horizontal line indicates the median, and whiskers indicate the 5th and 95th percentiles. Concentrations are shown on a logarithmic scale in CFU L^{-1}.

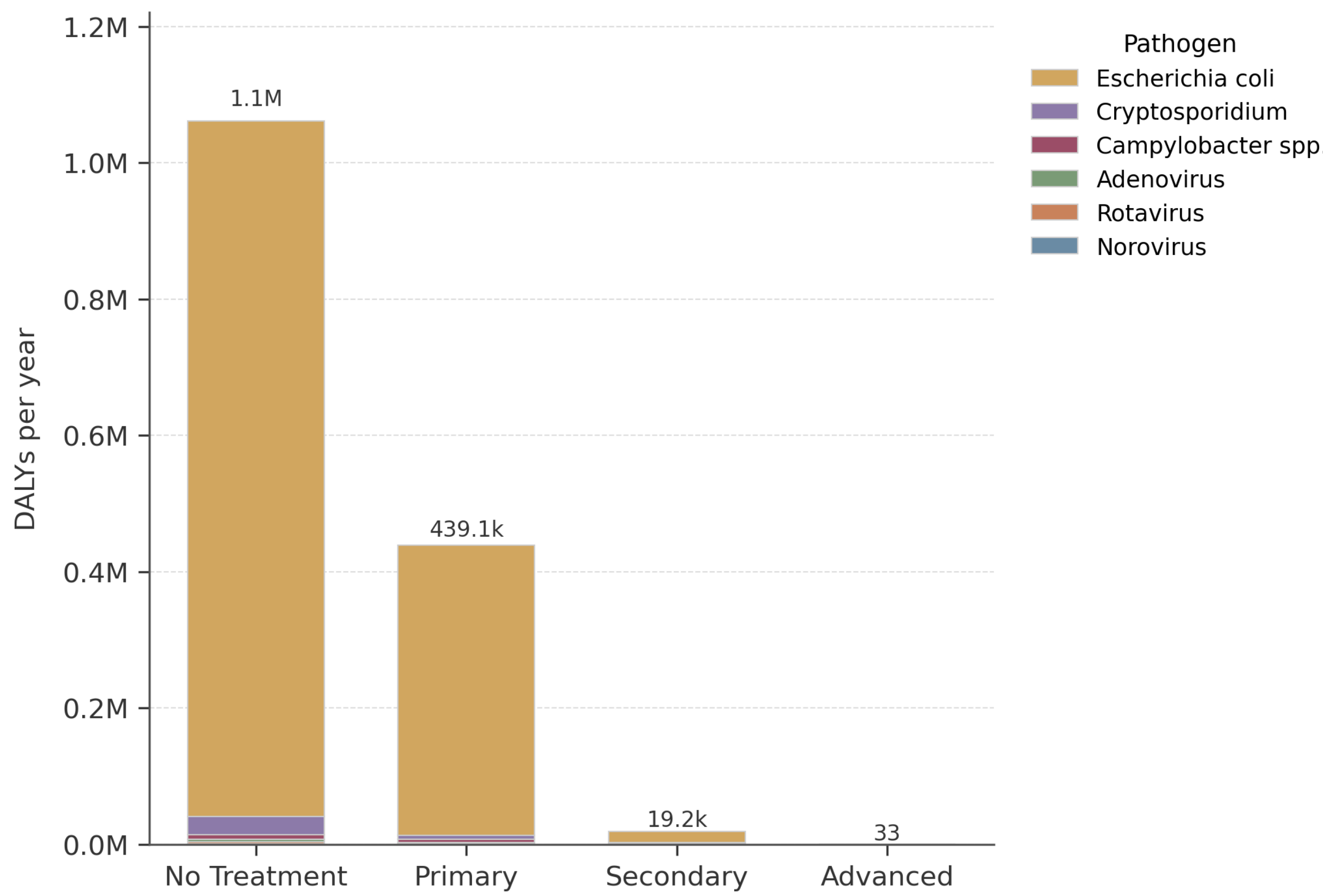

Figure A17: Pathogen-specific contributions to total annual DALYs across wastewater treatment scenarios. Stacked bars show the summed DALY burden attributable to each pathogen under each treatment scenario, with bar heights representing the total national annual health burden.

Effluent sensitivity

We tested the effects of the effluent concentrations by systematically change the effluent concentrations (Figure A18).

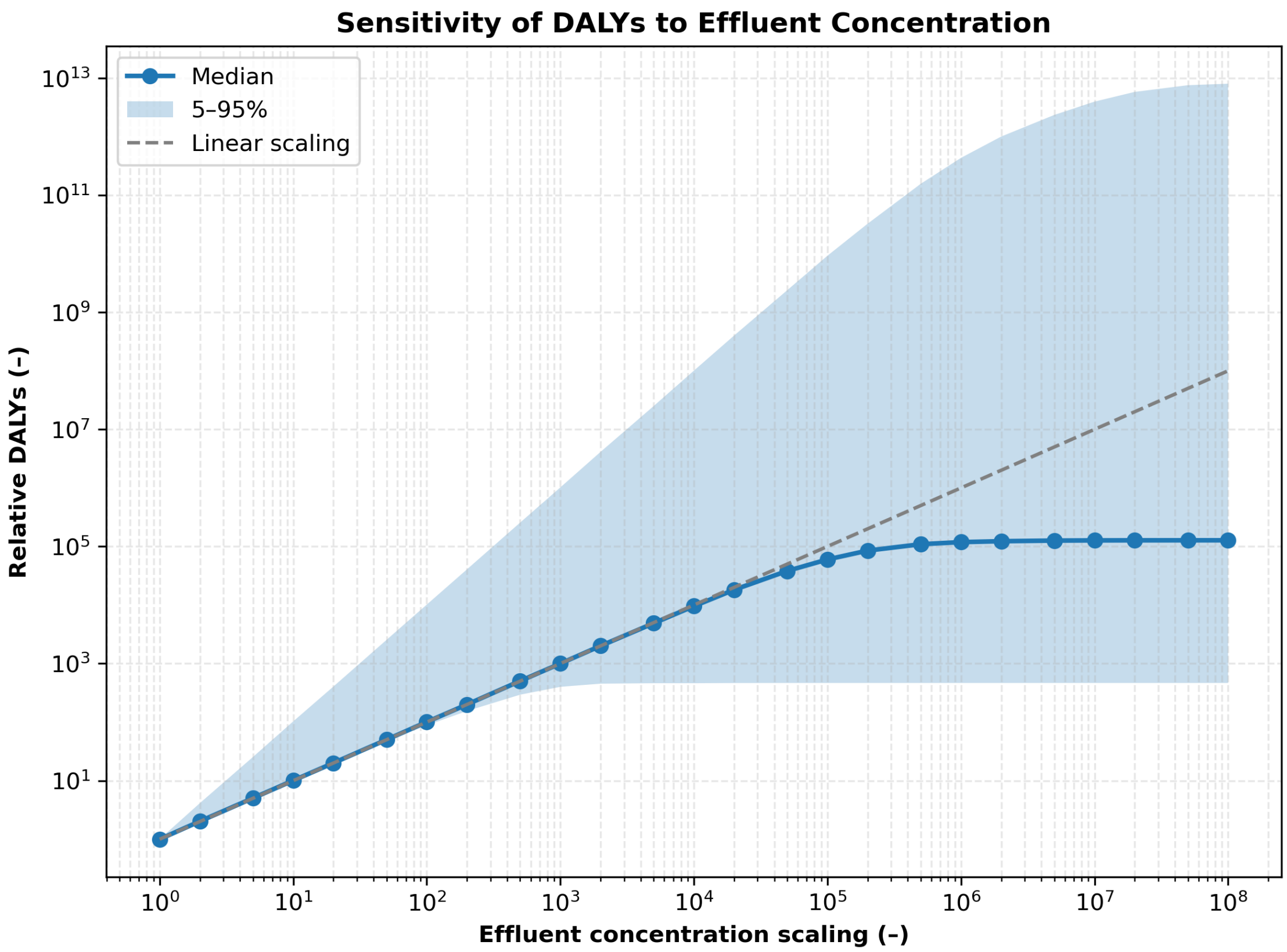

Figure A18: Sensitivity of health impacts to pathogen concentrations. Effluent concentrations are scaled over eight orders of magnitude relative to the baseline scenario, and the resulting relative DALYs are shown as the median (points) and the 5–95 % uncertainty range (shaded area). The dashed line indicates linear scaling for reference.
